# Dehydrogenative coupling of 4-substituted pyridines mediated by a zirconium(ii) synthon: reaction pathways and dead ends†
†Electronic supplementary information (ESI) available: Includes experimental details and characterization data of all new compounds, synthetic protocols, spectral data, X-ray crystallographic information (CIF), and xyz-files of the DFT-optimized geometries. CCDC 1826625–1826628. For ESI and crystallographic data in CIF or other electronic format see DOI: 10.1039/c8sc01025k


**DOI:** 10.1039/c8sc01025k

**Published:** 2018-05-16

**Authors:** Lukas S. Merz, Hubert Wadepohl, Eric Clot, Lutz H. Gade

**Affiliations:** a Anorganisch Chemisches Institut , Universität Heidelberg , Im Neuenheimer Feld 270 , 69120 Heidelberg , Germany . Email: lutz.gade@uni-heidelberg.de; b Institut Charles Gerhardt Montpellier , UMR 5253 CNRS-UM-ENSCM , Université de Montpellier , Place Eugène Bataillon, Bât 15, cc1501 , 34095 Montpellier Cedex 5 , France . Email: eric.clot@umontpellier.fr

## Abstract

The mechanism of the reductive homocoupling of pyridine derivatives mediated by the Zr^II^ synthon [(PNP)Zr(η^6^-toluene)Cl] (**1**) has been investigated. Selective transformation into three different types of product complexes has been observed, depending on the N-heterocyclic substrate employed: the bipyridyl complexes **3-R** (R = Me, Et, ^*t*^Bu, Bn, Ph, CHCHPh), which are the homocoupling products, the η^2^-((4-dimethylamino)pyridyl) complex **4** as well as the bis(isoquinolinyl) complex **5**. By deuterium labelling experiments the participation of the ligand backbone in the pyridine coupling reaction *via* potential cyclometallation steps was ruled out. Based on DFT modelling of the possible reaction sequences a reaction mechanism for the coupling sequence could be identified. The latter is initiated by a reductive *syn* C–C coupling rather than based on an initial C–H activation of the pyridine substrate.

## Abstract 2

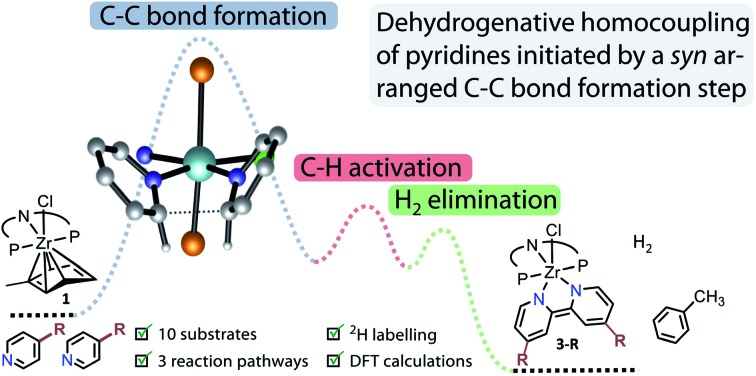
The mechanism of the reductive homocoupling of pyridine derivatives mediated by the Zr^II^ synthon [(PNP)Zr(η^6^-toluene)Cl] (**1**) has been investigated.

## Introduction

Early transition metal complexes are known to induce characteristic types of C–H activations^1^–^15^ which complement the analogous reactivity patterns established for their d-electron rich late transition metal counterparts.^16^–^25^ Cyclometallations may be viewed as particularly favourable variations of this type of reactivity^26^–^34^ and in their simplest form give rise to three-membered metallacycles. In this context group 4 metal compounds were found to activate pyridine to give pyridyl complexes incorporating η^2^-N,C-metallazirine moieties.^35^–^45^ Generally, the formation of these η^2^-pyridyl ligands may occur in two ways: either an alkyl substituent in M^IV^ alkyl complexes abstracts a proton from a coordinated pyridine molecule to generate the metallacycle^33^,^38^–^43^ or an M^II^ species, commonly generated *in situ* from the corresponding M^IV^ complexes, activates the pyridine molecule through oxidative addition with concomitant formation of an additional hydrido ligand.^35^,^36^,^46^ The former activation pattern was most recently utilized by Mindiola, Baik and coworkers in a detailed study which demonstrated that the transitory generation of a titanaazirine species from the benzopyridines, quinoline, and isoquinoline could result in the C–C coupling (as well as ring opening) of two substrate molecules.^47^

**Chart 1:**
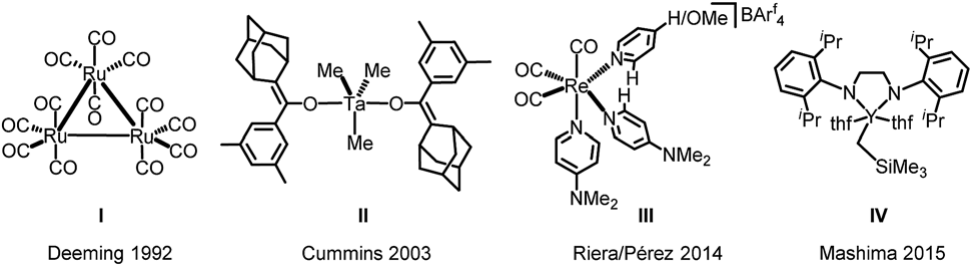
Selection of transition metal complexes mediating the stoichiometric reductive coupling of pyridine to 2,2′-bipyridine (II: reaction under a H_2_ atmosphere; III: KN(SiMe_3_)_2_ and AgOTf are needed additionally).

It is notable that despite its broad application as a ligand in coordination chemistry,^48^–^51^ the generation of 2,2′-bipyridine directly from pyridine is still in its infancy especially since transition metal catalysed cross-coupling reactions generally require 2-halopyridines and 2-metallated pyridines.^52^ Without prior functionalization of the pyridine cycle, the direct synthesis of 2,2′-bipyridines is hitherto almost entirely restricted to the application of heterogeneous catalysts such as RANEY® nickel and Pd/C.^53^–^56^ To our knowledge, only three homogeneous catalytic systems, using di- or trinuclear Ru- or Ru/Co-complexes,^57^–^59^ have been reported that are capable of catalysing the homocoupling of pyridines. Furthermore, even examples for the stoichiometric transformation of pyridine to 2,2′-bipyridine are rare and have only been described for a limited number of substrates (*cf.*Chart 1).^60^–^63^ The reaction pathways involved remain incompletely understood.

Recently, we reported the synthesis of the ^Cbz^(PNP)ZrCl(η^6^-arene) complex **1**, in which the η^6^-toluene ligand displays a puckered arrangement of the arene ring similar to other early TM arene complexes.^64^–^68^ This indicates a significant arene-1,4-diido character of the tolyl ring, and the zirconium atom in this complex is therefore to be assigned to the oxidation state +4.

**Scheme 1:**
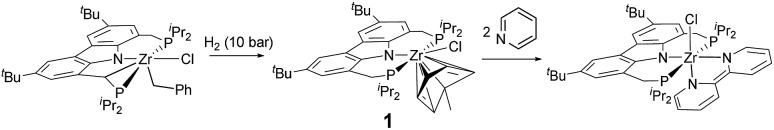
Hydrogenolytic formation of Zr^II^ synthon **1** from a cyclometallated benzyl complex and its reaction with two molar equivalents of pyridine.

However, complex **1** can be regarded as a Zr^II^ synthon, since the displacement of a neutral toluene molecule would provide access to a transient Zr^II^ species. This could be demonstrated *inter alia* by the reductive coupling of pyridine to form a dianionic bipyridyl ligand coordinated to the zirconium centre (Scheme 1).^69^ The latter raised the question about the scope of this transformation, on the one hand, and the reaction mechanism which leads to this dehydrogenative C–C coupling of two N-heterocycles. The latter was deemed to involve a complex sequence of bond scission and formation steps within the coordination sphere of the metal atom. In this work new light will be shed on this type of reaction highlighting the specific preconditions for such a transformation as well as the unproductive dead ends of the underlying network of reaction steps.

## Discussion

To obtain the first experimental mechanistic clues for the zirconium mediated reductive coupling of pyridine, we probed the substrate scope for this transformation using the isolated η^6^-arene complex **1**.^69^

**Scheme 2:**
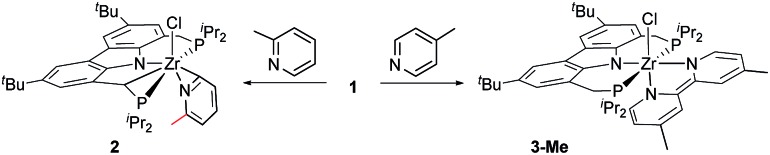
Reactivity of arene complex **1** with 2-picoline in contrast to the reactivity with 4-picoline (product **2** could not be purely isolated; observed *via*^1^H NMR and X-ray analysis).

In the first assay, monitored *in situ* by ^31^P NMR spectroscopy, four methyl substituted pyridines were tested (Table 1) and it was found that the previously observed reductive coupling reaction strongly depended on the substitution pattern. For pyridines methylated in the *meta*-position, unselective conversion into a variety of unidentifiable products was observed (Table 1, entries 1–2), 2-picoline underwent cyclometallation at the metal but no subsequent C–C coupling occurred (Table 1, entry 3 and Scheme 2, left) whereas 4-picoline was transformed to the expected dimethylated bipyridine (Table 1, entry 4 and Scheme 2, right).

**Table 1:** Dehydrogenative coupling of differently substituted pyridines mediated by **1**^*a*^

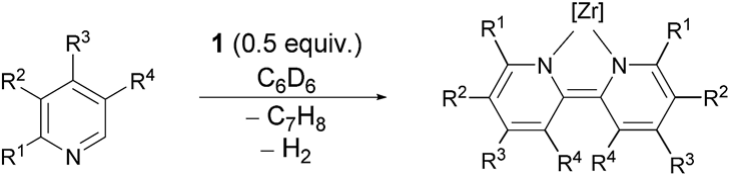				
Entry	Substrate	T^*c*^ [°C]	Time [h]	Ratio A:B^*d*^ ^*e*^
1	R^2^ = Me, R^1,3,4^ = H	60	16	—^*b*^
2	R^2,4^ = Me, R^1,3^ = H	80	48	—^*b*^
3	R^1^ = Me, R^2–4^ = H	100	16	66:34
4	R^3^ = Me, R^1,2,4^ = H	60	16	80:20

^*a*^The reactions were carried out in NMR tubes fitted with J. Young valves with 20 mg (26 μmol, 1.0 equiv.) of **1** with 2.0 equivalents of the corresponding pyridine.

^*b*^Unselective conversion to unidentified products was observed.

^*c*^Temperature was gradually increased from RT to either 60, 80 or 100 °C in case no reaction occurred.

^*d*^A = main product; B = unidentified by-product(s); judged by ^31^P spectroscopy.

^*e*^Starting complex **1** was fully consumed in the stated reaction time; judged by ^31^P NMR spectroscopy.

For the 2-picoline substrate the observation of two doublet resonances in the ^31^P{^1^H} NMR spectrum (*δ*(^31^P) [ppm] = 18.2 (d, *J*_PP_ = 25.4 Hz), –1.2 (d, *J*_PP_ = 25.4 Hz)) indicated the cyclometallation of one of the methylene bridges of the ^Cbz^PNP pincer ligand.^70^ The result of a single crystal X-ray structure analysis confirmed the identity of complex **2** as an η^2^-pyridyl species (see ESI†). The results obtained for 2-, 3- and 4-substituted methyl pyridines indicated that the reductive C–C coupling reaction mediated by η^6^-arene complex **1** proceeded exclusively with 4-substituted pyridines.

The formation of the dianionic bipyridine ligand in the reaction of **1** with 4-picoline was accompanied by a change of colour from brown to deep purple and conversion to **3-Me** was completed after 16 h at 60 °C (*cf.*Scheme 2 and Table 1, entry 4). The reaction product was identified by ^31^P{^1^H} NMR spectroscopy (*δ*(^31^P{^1^H}) = 23.4 ppm (bs)) as well as a characteristic set of signals in the ^1^H NMR spectrum. In the case of **3-Me**, this set consists of three different doublet and two singlet signals spread over a range of 5 ppm representing the six protons of the bipyridyl ligand [*δ*(^1^H) = 9.18 (d, *J*_HH_ = 7.1 Hz, 1H); *δ*(^1^H) = 7.31 (bs, 1H); *δ*(^1^H) = 6.33 (s, 1H); 6.31 (s, 1H); *δ*(^1^H) = 4.87 (d, *J*_HH_ = 7.0 Hz, 1H); 4.29 (d, *J*_HH_ = 7.0 Hz, 1H) ppm].

To further investigate the scope of the coupling reaction, a series of 4-substituted pyridines was reacted with complex **1** (*cf.*Table 2). A general reactivity trend emerged in this study: pyridine substrates with reduced electron density in the aromatic ring did not undergo the reductive coupling reaction (*cf.*Table 2, entry 1 + 2) while the reaction with an electron-rich pyridine resulted in the formation of the coupled heterocycle along with significant amounts of side products, precluding the isolation of the respective bipyridyl complex (*cf.*Table 2, entry 10). However, substrates with similar electronic properties to the unsubstituted pyridine were readily transformed to the desired bipyridyl ligand. These included a variety of alkyl as well as sp^2^ substituted pyridines (Table 2, entries 3–9).

**Table 2:** Expanding the substrate scope for the dehydrogenative coupling of 4-substituted pyridines mediated by complex **1**

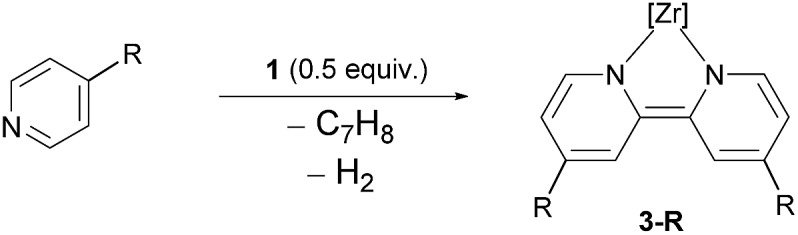				
Entry	Substrate	T [°C]^c^	Time [h]	Isolated yield [%]
1	R = CF_3_	80	24	—^*a*^
2	R = CN	50	24	—^*a*^
3	Pyridine-d_5_	50	19	21
4	R = Me	60	16	18
5	R = Et	50	19	25
6	R = ^*t*^Bu	50	19	34
7	R = CH_2_Ph	50	19	42
8	R = Ph	60	19	59
9	R = CHCHPh	50	19	44
10	R = OMe	50	19	—^*a*^
11	R = NMe_2_	25	20	42

^*a*^Unselective conversion to unidentified products was observed.

As expected, all bipyridyl complexes possess very similar properties: they are deeply coloured in the solid state and in solution, highly sensitive towards oxygen and moisture, and all display a characteristic set of proton resonances for the newly formed bipyridine ligand between 9.2 and 4.5 ppm (in addition to the resonances of their respective substituents). The extreme solubility of all complexes in hydrocarbons, including *n*-pentane and *n*-hexane, led to significant discrepancies between the observed degree of conversion and the actual isolated yields in the purification based on washing or recrystallization.

**Fig. 1:**
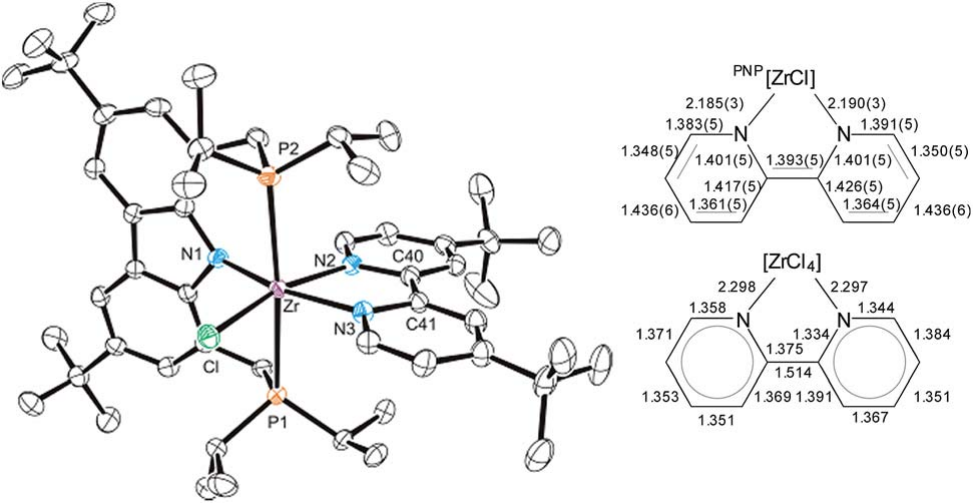
Left: molecular structure of **3-*^t^*Bu** (hydrogen atoms were omitted for clarity, ellipsoids set at 50% probability). Selected bond lengths [Å] and angles [°] for **3-*^t^*Bu**: Zr–Cl 2.4931(9), Zr–P1 2.7490(10), Zr–P2 2.7436(10), Zr–N1 2.243(3), Zr–N2 2.190(3), Zr–N3 2.185(3), Cl–Zr–P1 87.16(3), Cl–Zr–P2 91.52(3), P2–Zr–P1 175.39(3), N1–Zr–Cl 105.42(8), N1–Zr–N2 88.79(11), N2–Zr–N3 71.09(11), N3–Zr–Cl 94.48(8), N2–Zr–P1 87.99(8), N2–Zr–P2 94.38(8). Right: comparison between bond lengths: bipy^2–^ of **3-*^t^*Bu** to bipy^0^.^71^

For **3-*^t^*Bu**, the crystal structure was determined from single crystals grown by cooling saturated *n*-pentane solutions to –40 °C (*cf.*Fig. 1). As expected, the molecular structure displays octahedral coordination geometry with the ^Cbz^(PNP) ligand in a meridional coordination mode while the other three coordination sites are occupied by the chlorido and the bipyridyl ligand. The molecule is approximately *C*_s_ symmetric along the equatorial plane of the octahedron, which is in agreement with the symmetry derived from the NMR signal patterns in solution.

The dianionic character of the bipyridyl ligand becomes apparent upon comparison of its C–C and Zr–N bond lengths with those of a zirconium bipy^0^ complex reported in the literature (*cf.*Fig. 1).^71^ In the molecular structure of **3-*^t^*Bu**, contracted Zr–N bond lengths (Zr–N2 2.190(3); Zr–N3 2.185(3)) as well as alternatingly shortened C–C bond lengths within and between the two pyridine rings were found, suggesting the (partial) loss of aromatic character in the bipyridyl ligand and the localization of the double bonds. The presence of a bipy^2–^ ligand is further corroborated by two broad absorption bands in the UV/Vis spectrum that can be attributed to the intraligand π–π* transitions of the bipy^2–^ ligands.^72^–^74^

**Scheme 3:**
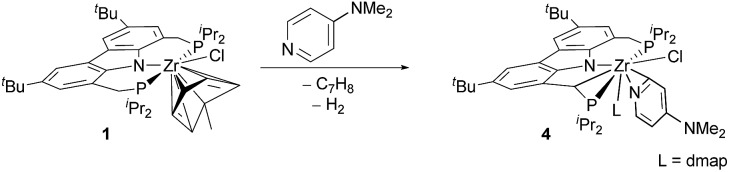
Conversion of η^6^-arene complex **1** with 4-(*N*,*N*-dimethylamino)pyridine to cyclometallated η^2^-pyridyl complex (L = DMAP).

As mentioned above, an increase of electron density in the pyridine substrate tended to give rise to an inseparable product mixture (*cf.*Table 2, entry 10). However, further increase in the electron donating character of the substituent in the 4-position (in this case an *N*,*N*-dimethylamine substituent in 4-(dimethylamino)pyridine (DMAP)) led to the selective conversion into cyclometallated pyridyl complex **4** (*cf.*Table 2, entry 11 and Scheme 3). As for the analogous complex **2**, the ^1^H, ^13^C and ^31^P NMR spectra of **4** indicated a C–H activated ligand backbone of the PNP pincer.

The ^1^H NMR spectrum of the isolated product, in particular, illustrated the activation of the ligand backbone. Due to the loss of one proton in the CH_2_-linkers, both linker units give rise to a characteristic signal pattern: three separate signals with the relative intensities of one proton each (*δ*(^1^H) [ppm] = 4.95 (dd, *J*_HH_ = 16.0 Hz, *J*_HP_ = 4.4 Hz), 3.60 (dd, *J*_HH_ = 14.9 Hz, *J*_HP_ = 14.9 Hz), 2.39 (m)).

**Fig. 2:**
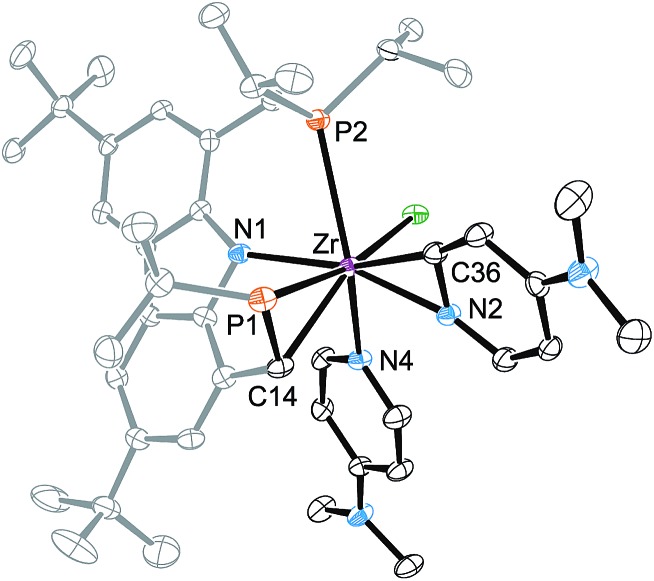
Molecular structure of **4**. Hydrogen atoms and one isopropyl group were omitted for clarity, ellipsoids set at 50% probability. Selected bond lengths [Å] and angles [°] for **4**: Zr–Cl 2.5597(7), Zr–P1 2.7280(7), Zr–P2 2.8220(7), Zr–N1 2.260(2), Zr–N2 2.204(2), Zr–N4 2.395(2), Zr–C14 2.412(3), Zr–C36 2.226(3), Cl–Zr–P1 168.16(2), Cl–Zr–P2 75.27(2), P2–Zr–P1 92.90(2), N1–Zr–Cl 99.18(5), N1–Zr–N2 162.55(8), N2–Zr–N4 86.42(7), N2–Zr–Cl 93.01(6), N2–Zr–P1 93.82(6), N2–Zr–P2 122.69(6).

To establish the structural details of **4**, a single-crystal X-ray structure analysis was carried out (Fig. 2). It confirmed the presence of the cyclometallated ligand backbone of the ^Cbz^(PNP) pincer ligand causing a deformation of the natural *meridional* coordination mode drawing the coordinating phosphines towards each other. As in compound **2**, the pyridine substrate had undergone *ortho* C–H activation to form an η^2^-pyridyl unit; however, in the case of **4** an additional substrate molecule is coordinated to the metal atom. As opposed to the solid-state structure of **3-*^t^*Bu** (Fig. 1) the coordination mode for the ^Cbz^PNP ligand in **4** is better described as distorted facial than as meridional with the remaining three coordination sites being inhabited by the η^2^-pyridyl moiety, and the chlorido and the DMAP ligand.

**Chart 2:**
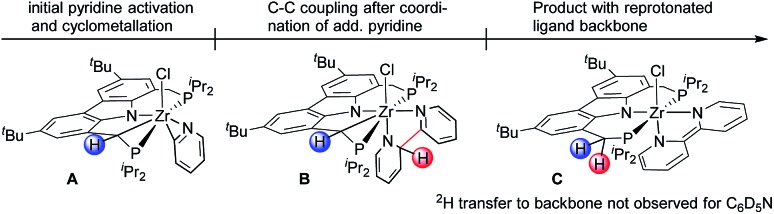
Illustration of essential intermediates envisioned for the reductive coupling of pyridine involving a cyclometallated ligand backbone.

Given the formation of the η^2^-pyridyl complexes **2** and **4**, there appeared to be a pronounced tendency of the ^Cbz^(PNP) ligand in combination with early TM to undergo cyclometallation reactions.^70^ It therefore was conceivable that the activation of the ligand backbone played a part in the reaction pathway resulting in the reductive coupling of pyridines. In this context, an initial C–H activation step forming an (η^2^-pyridyl)zirconium(iv) hydride species appeared to be possible.^36^ Such a potential intermediate in turn could activate the ligand backbone to provide **A** through elimination of H_2_ (*cf.*Chart 2). After the coordination of a second substrate molecule, C–C coupling could take place forming complex **B** while the ^Cbz^(PNP) ligand would be restored in the last step through an H-atom transfer from the coupled bipyridyl ligand to generate complex **C**.

**Fig. 3:**
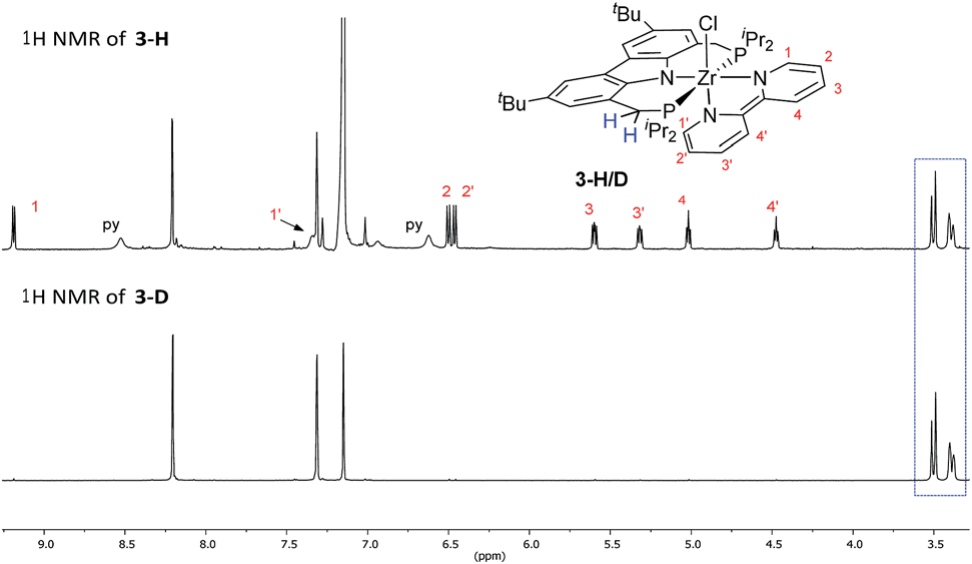
Comparison of ^1^H NMR (600.13 MHz, 298 K) spectra of **3-H** with **3-D**. The two doublet signals marked by the blue rectangle belong to the methylene bridges of the coordinated ^Cbz^(PNP) ligand. Consequently, there is no indication of deuteration of these positions.

Such a reaction pathway would imply hydrogen atom exchange between the methylene hydrogen atoms in the PNP pincer and the pyridine substrate. Thus, in order to probe this specific consequence of such a mechanism the coupling reaction was repeated with pyridine-d_5_ in benzene-d_6_. This did not lead to deuterium incorporation into the ligand backbone (*cf.*Fig. 3) which allowed us to exclude such a cyclometallation step involving the PNP ligand backbone. It also implied that the isolated complexes **2** and **4** were the result of a competitive reaction pathway not leading to the formation of a bipy^2–^ complex but rather representing a dead end for this type of transformation.

**Scheme 4:**
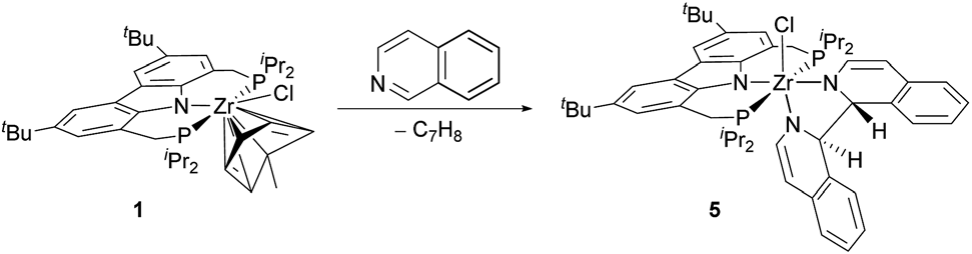
Reaction of Zr^II^ synthon 1 with isoquinoline.

In the course of the substrate screening a remarkable new reactivity pattern was observed. When isoquinoline was reacted with Zr^II^ synthon **1**, the isoquinoline indeed underwent a C–C coupling reaction yielding bisisoquinoline complex **5**. However, the NMR data of the product indicated that the coupling step occurred without the loss of the two hydrogen atoms at the bridge carbons^75^ as it was the case for several of the 4-substituted pyridine substrates (Scheme 4).

**Fig. 4:**
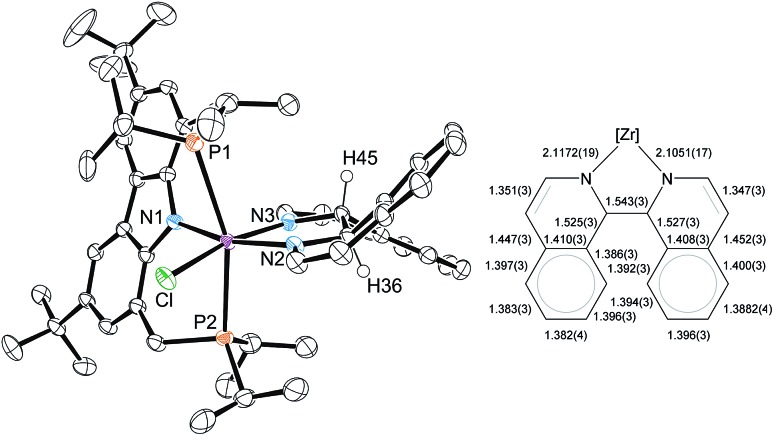
Left: molecular structure of **5** (only one of the two independent molecules is shown; most hydrogen atoms were omitted for clarity, ellipsoids set at 50% probability). Selected bond lengths [Å] and angles [°] for **5** (values in square brackets refer to the second independent molecule): Zr–Cl 2.5130(5) [2.5062(5)], Zr–P1 2.7506(6) [2.7425(5)], Zr–P2 2.7262(5) [2.7234(5)], Zr–N1 2.2450(16) [2.2484(15)], Zr–N2 2.1051(16) [2.1122(16)], Zr–N3 2.1179(17) [2.1215(16)], Cl–Zr–P1 85.940(19) [86.674(18)], Cl–Zr–P2 85.253(18) [84.928(18)], P2–Zr–P1 160.277(17) [157.902(17)], N1–Zr–Cl 103.34(4) [104.13(4)], N1–Zr–N2 162.90(7) [163.25(6)], N2–Zr–N3 75.02(6) [74.84(6)], N3–Zr–Cl 166.50(5) [165.05(4)], N2–Zr–P1 96.26(5) [97.13(5)], N2–Zr–P2 101.77(5) [103.51(5)]. Right: illustration of C–C bond lengths in the bis(isoquinoline) ligand of the depicted molecule.

An X-ray diffraction study of **5** established the details of its molecular structure (Fig. 4). The molecular structure revealed a complex with distorted octahedral coordination geometry, in which a bisisoquinoline ligand occupies two coordination sites. The C1–C1′ bond length of the bisisoquinoline ligand is consistent with a single bond between the separate isoquinoline moieties. Moreover, the solid-state structure confirmed that no formal elimination of dihydrogen had occurred and that the remaining hydrogen atoms at the bridge carbons are arranged in the *anti*-disposition. In combination with the twisted isoquinoline moieties, these hydrogen atoms break the *C*_s_ symmetry generally found in complexes lacking an activated ligand backbone. As a result, the ^1^H and ^31^P NMR spectra show the signal patterns corresponding to a *C*_1_ symmetric complex.

### DFT modelling of the reductive coupling of 4-substituted pyridines

**Fig. 5:**
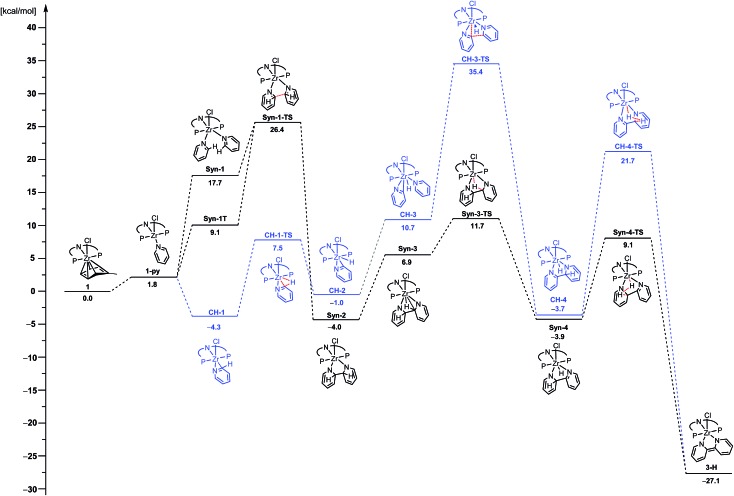
Calculated free energies (kcal mol^–1^) of key intermediates and transition states of two envisaged reaction sequences at 1 bar and 60 °C. Blue represents a pathway with an initial C–H activation step, whereas black depicts a pathway with the C–C coupling step of inactivated pyridine as the first transformation (all structures except **Syn-1T** have been computed as singlet species; all structures but **1-py**, **Syn-1** and **CH-1** are Zr^IV^ species).

As group 4 complexes are known to promote the C–H activation of pyridines, the formation of an η^2^-pyridyl complex was initially assumed as the logical Zr^IV^ intermediate in the mechanism to form complexes **3-R**. However, there are reports of a high propensity of Zr^II^ species, such as the one generated from **1** on elimination of toluene, to facilitate C–C coupling reactions. Exemplary work has been reported by Rosenthal and co-workers with utilization of zirconacenes.^76^–^81^ Due to this reactivity of low-valent zirconium species and the occurrence of complex **5**, the possibility of the C–C coupling of the substrates prior to their C–H activation could not be neglected as a viable reaction mechanism. Hence, two general reaction sequences were envisaged: (i) the initial C–H activation of one pyridine molecule, as it has previously been proposed,^61^ and (ii) the C–C bond formation as the first reaction step. To distinguish between these sequences of events, DFT (PBE0) modelling for the two alternative reaction pathways was performed.^82^ The calculated reaction energy profiles for the reductive C–C coupling of pyridine are summarized in Fig. 5. Both pathways start with the extrusion of a neutral toluene molecule by pyridine to form the Zr^II^ species **1-py** in an almost thermoneutral transformation.

In the case of the C–H activation pathway (Fig. 5, blue), isomerisation to a geometry, **CH-1**, with the C–H bond at the 2-position ready to be cleaved is exergonic (Δ*G* = –6.1 kcal mol^–1^). The actual C–H bond cleavage is effective through **CH-1-TS** with an activation barrier of Δ*G*^‡^ = 11.8 kcal mol^–1^, leading to the η^2^-N,C-pyridyl intermediate **CH-2**. After the endergonic coordination of an additional pyridine molecule to yield **CH-3**, the formation of the new C–C bond between the two heterocycles is effective through **CH-3-TS** with an activation barrier of Δ*G*^‡^ = 24.7 kcal mol^–1^ from **CH-3**. The resulting product of this C–C coupling is barely less stable than the initial complex **CH-1** (Δ*G* = +0.6 kcal mol^–1^). The final product of the transformation, **3-H**, is obtained when H_2_ is formed through **CH-4-TS** with an activation barrier of Δ*G*^‡^ = 25.4 kcal mol^–1^ from **CH-4**. Overall, the energy barrier to overcome is Δ*G*^#^ = 39.7 kcal mol^–1^. This elevated activation barrier is consistent with a recent study by Mindiola, Baik and co-workers. They computed the cleavage of an η^2^-N,C-quinolyl unit [bonded as titanaaziridine moiety] with subsequent nucleophilic attack on a quinoline molecule at similar elevated energies of 27.9 to 40.1 kcal mol^–1^ (depending on the substrate and spin state).^47^ In light of our results and the congruence with previous findings, it was concluded that the reductive coupling reaction of pyridine unlikely proceeds along this reaction pathway.

**Chart 3:**
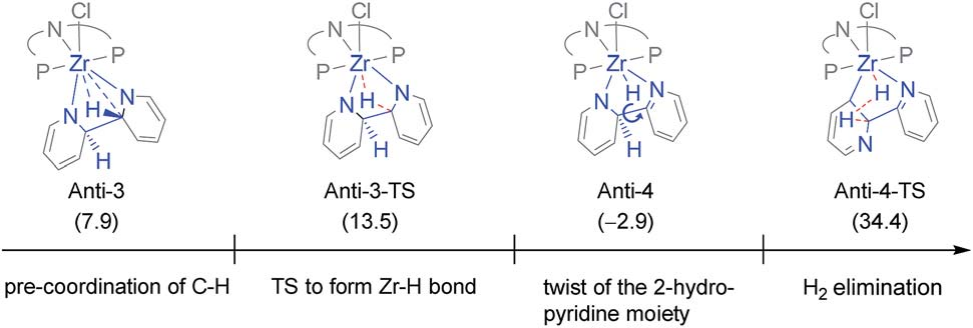
Visualization of the disfavoured H_2_ elimination in the case of the *anti* C–C coupling sequence. Values in parentheses are calculated free energies in kcal mol^–1^ in reference to η^6^-arene complex **1** (**Anti-4-TS**: due to the twist around the py–py axis, the negative charge is located at the C6 carbon atom, which coordinates to the zirconium metal centre).

Hence, the focus was shifted towards an initial C–C coupling step of two pyridine molecules. Two reaction sequences are possible: the two pyridine moieties could undergo C–C coupling with the hydrogen atoms in the 2-positions pointing to (i) the same (*syn*) or (ii) to the opposite (*anti*) direction of the formed bipyridine plane. The latter was eliminated as H_2_ elimination from the *anti*-coupled intermediate, **Anti-3**, appeared unlikely (*cf.*Chart 3).^83^ Even though the first C–H activation to transfer one hydrogen atom onto Zr is easy (Δ*G*^‡^ = 5.6 and Δ*G* = –10.8 kcal mol^–1^), forming **Anti-4**, the subsequent H-transfer to form H_2_ is associated with a high lying TS (**Anti-4-TS**). In addition, the TS for rotation around the central C–C bond to reach, from **Anti-4**, a geometry more suited to C–H bond cleavage in **Anti-4-TS** could not be found. Therefore, the energy difference between **Anti-4-TS** and **Anti-4**, Δ*G*^‡^ = 37.3 kcal mol^–1^, is a lower limit of the actual activation barrier. This is too high a value to be representative of the situation observed experimentally.

**Fig. 6:**
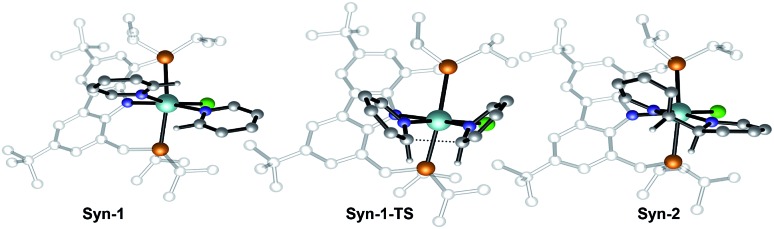
Optimized geometries of the extrema located along the reaction pathway for the C–C bond formation.

**Fig. 7:**
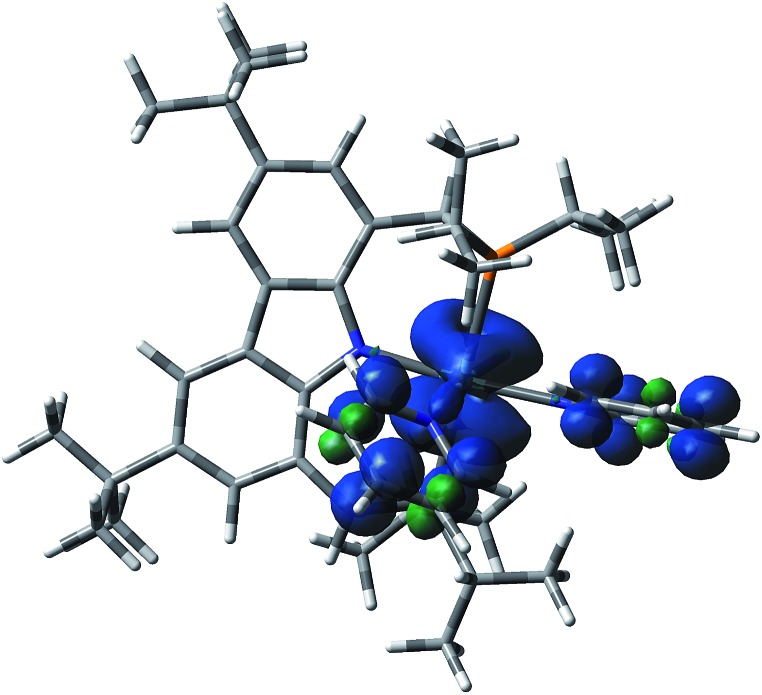
Visualization of the spin density in **Syn-1T**, showing the spin density in the *ortho* and *para* carbon atoms of the coordinated pyridines.

**Fig. 8:**
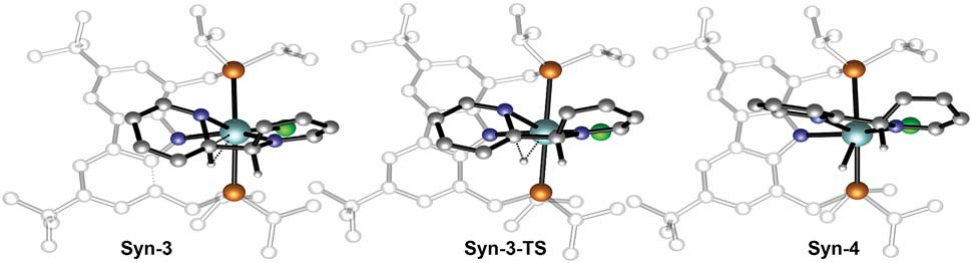
Optimized geometries of the extrema located along the reaction pathway for the hydrogen transfer from the C–C coupled 2*H*,2′*H*-[2,2′-bipyridine]-1,1′-diide ligand to the zirconium central atom.

Therefore, attention was focused on the *syn* C–C coupling reaction sequence (Fig. 5, black). After the almost thermoneutral substitution of toluene by pyridine and the concomitant formation of a low-valent Zr^II^ species, the coordination of a second pyridine molecule generates the precursor of the C–C coupling process, **Syn-1**, at Δ*G* = 17.7 kcal mol^–1^ with respect to the starting reactants. The exergonic formation of the C–C bond leading to **Syn-2** (Δ*G* = –21.7 kcal mol^–1^ from **Syn-1**) is effective through **Syn-1-TS** with Δ*G*^‡^ = 8.7 kcal mol^–1^ (Fig. 6) and transforms the Zr^II^ species, **Syn-1**, into the Zr^IV^ species, **Syn-2**. Considering this, two spin states are possible: **Syn-1** or **Syn-1T** and **Syn-1-TS** (singlet or triplet). A geometry for **Syn-1** in the triplet state, **Syn-1T**, could be located on the potential energy surface at Δ*G* = 9.1 kcal mol^–1^ with respect to **1**. Fig. 7 shows the spin density for **Syn-1T** and clearly highlights significant accumulation of spin density on both the *ortho* and *para* carbon atoms of the coordinated pyridine ligands. This electronic pattern is computed to be more stable than the singlet state **Syn-1** and supports electron transfer from Zr to the coordinated pyridine before the actual C–C coupling step. However, attempts to locate a triplet transition state similar to **Syn-1-TS** failed as the same spin density on the *ortho* carbon atoms would prevent formation of the C–C bond, indicating the limitations of the computational strategy adopted in this paper. Subsequent isomerization to **Syn-3** to position the C–H bond close to Zr is endergonic (Δ*G* = 10.9 kcal mol^–1^) but leads to an easy C–H bond cleavage through **Syn-3-TS** (Δ*G*^‡^ = 4.8 kcal mol^–1^, Fig. 8). The product of this C–H activation, **Syn-4**, lies at the same energy as the product of C–C *syn* coupling **Syn-2**.

**Fig. 9:**
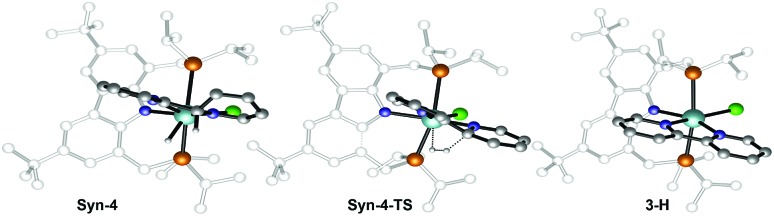
Optimized geometries of the extrema located along the reaction pathway for the hydrogen elimination from Syn-4 to form bipyridyl complex 3-H.

The second C–H bond cleavage is achieved through **Syn-4-TS**, in a σ-bond metathesis with Δ*G*^‡^ = 13 kcal mol^–1^ (Fig. 9). The last transformation is strongly exergonic and forms the product **3-H**. Overall the energy barrier to overcome in order to form **3-H** along the reaction pathway depicted in black in Fig. 5 is 26.4 kcal mol^–1^, associated with the TS for the C–C bond formation between two coordinated pyridine ligands.

Such a value is in very good agreement with the observed experimental conditions in terms of reaction time and temperature. To further probe the validity of this mechanism, the theoretical kinetic isotope effect (KIE) of the rate determining step for the **Syn** and the **CH** reaction sequences was modelled through normal coordinate analysis of the DFT reactant and the transition state structures (reactant (**Syn-1**/**CH-3**) → ts (**Syn-1-TS**/**CH-3-TS**)) by the Bigeleisen–Mayer approach.^84^–^86^ This resulted in the KIE(**Syn-1-TS**)_theor_ = 1.32 for the transformation from **Syn-1** to **Syn-2**, whereas a value of KIE(**CH-3-TS**)_theor_ = 0.92 was found for the conversion of **CH-3** to **CH-4**. The experimental KIE was evaluated through the reaction of ^Cbz^(PNP)ZrCl(η^6^-arene) complex **1** with an excess of both, pyridine and pyridine-d_5_, and determined to be KIE_exp_ = 1.29. The experimental value agrees well with KIE(**Syn-1-TS**)_theor_ and further corroborates that the dehydrogenative coupling of 4-substituted pyridines proceeds along the suggested *syn* C–C coupling sequence (*cf.*Fig. 5 – black).

### DFT modelling of the competitive reaction paths for 2-picoline, DMAP and isoquinoline

Based on deuteration experiments as well as DFT calculations, the reaction mechanisms involving a cyclometallated ligand backbone or an *anti*-arranged C–C coupling step were precluded as viable reaction pathways for the reductive coupling of pyridine and a variety of its 4-substituted analogues. Nevertheless, it was observed that selected substrates were converted into intermediates of the two aforementioned reaction pathways (*cf.*Schemes 3 and 4). To get more insight into these experimental observations, the computations for the crucial transition states found for the reductive coupling of pyridine were repeated with the respective substrate molecules, namely 2-picoline, DMAP and isoquinoline.

**Fig. 10:**
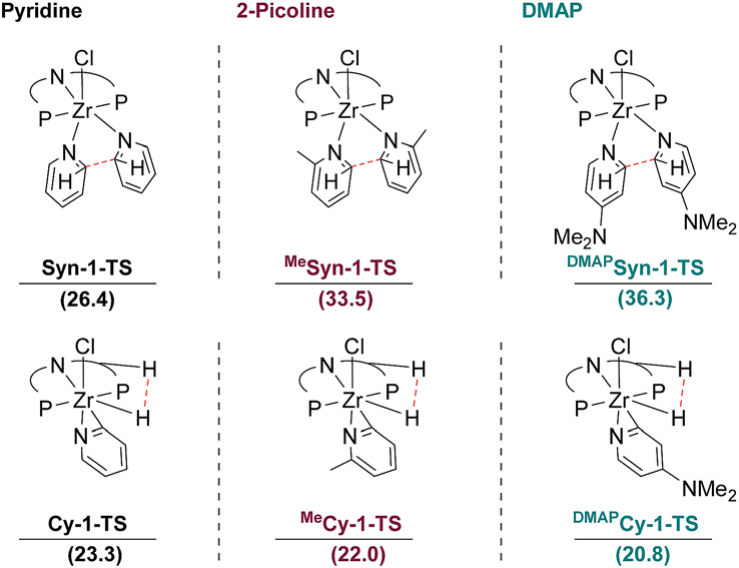
Comparison of the Gibbs free energies (kcal mol^–1^) of the TS for *syn* C–C coupling and H_2_ extrusion to form cyclometallated η^2^-pyridyl complexes: left for pyridine, middle for 2-picoline and right for DMAP.

For 2-picoline and DMAP, the initial *syn* C–C coupling transition states were modelled. The calculated activation barriers were significantly higher for 2-picoline (Δ*G*^‡^ = 15.3 kcal mol^–1^) and DMAP (Δ*G*^‡^ = 22.3 kcal mol^–1^) than the value obtained for pyridine (Δ*G*^‡^ = 8.7 kcal mol^–1^). As a consequence, the corresponding TS (**^Me^Syn-1-TS** and **^DMAP^Syn-1-TS**) lie at too high free energies with respect to the separated reactants to provide reactive pathways (Fig. 10).

In the case of pyridine, the reaction pathway involving a cyclometallated ligand was excluded based on the deuterium labelling experiments. Nevertheless, the transition state **Cy-1-TS** corresponding to the extrusion of H_2_ from **CH-2** leading to the cyclometallated η^2^-pyridyl complex **Cy-2** was computed to lie at Δ*G* = 23.3 kcal mol^–1^ with respect to the separated reactants (Fig. 10). The computed Gibbs free energies for **Syn-1-TS** (26.4 kcal mol^–1^) and **Cy-1-TS** (23.3 kcal mol^–1^) support preferred formation of **Cy-2** with respect to **Syn-2**, both lying at the same energy (Δ*G* = –4.0 kcal mol^–1^). However, the values of the Gibbs free energies for the two competing transition states are rather to one another.

Interestingly, the difference in energy between these two crucial transition states deciding the final outcome of the reaction is strongly influenced by the nature of the substituent on the pyridine. With 2-picoline and DMAP, the transition states, **^Me^Cy-1-TS** and **^DMAP^Cy-1-TS**, leading to the cyclometallated η^2^-pyridyl complex, **^Me^Cy-2** and **^DMAP^Cy-2**, lie at lower energy (22.0 and 20.8 kcal mol^–1^, respectively) compared to the unsubstituted case (Fig. 10). With the substituted pyridine rings there is thus a clear preference for the pathway leading to the cyclometallated η^2^-pyridyl complex in perfect agreement with the experimental observations. This situation is the result of both a more energy-demanding C–C coupling for the substituted heterocycles and an easier H_2_ extrusion upon cyclometallation.

With 2-picoline and DMAP, the cyclometallated η^2^-pyridyl complexes are dead-ends as further reaction with another equivalent of substituted pyridine is associated with high lying transition states. The activation barrier for C–C coupling from **^Me^Cy-2** and **^DMAP^Cy-2**, through **^Me^Cy-3-TS** and **^DMAP^Cy-3-TS**, are Δ*G*^#^ = 40.6 kcal mol^–1^ and Δ*G*^#^ = 34.3 kcal mol^–1^, respectively. The reactivity of 2-picoline can be traced back to a steric repulsion of the methyl substituents with the isopropyl groups of the ligand destabilizing **^Me^Syn-1-TS**. In contrast, the reactivity of DMAP is electronically induced due to a more facile activation of C–H bonds of electron rich arenes.^12^ Furthermore, other 4-substituted pyridines with sterically more demanding substituents were readily transformed to **3-R**, ruling out steric effects of the NMe_2_ substituent in DMAP.

**Fig. 11:**
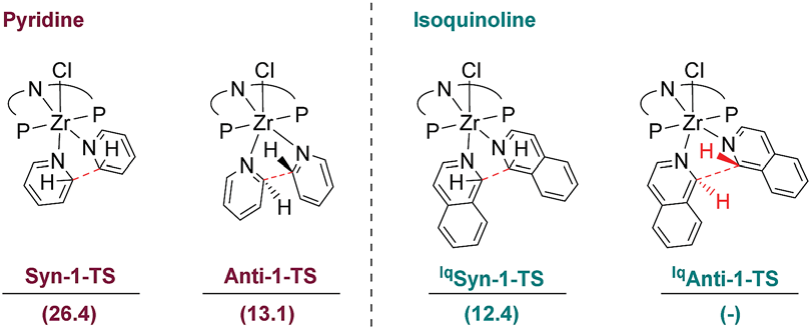
Gibbs free energies (kcal mol^–1^), relative to the separated reactants, for the *syn*- and *anti*-transition state for pyridine as well as isoquinoline.

Further attention was given to the competitive reaction path resulting in the *anti* C–C coupling of isoquinoline to form complex **5**. For this purpose, the previously described *syn*- and *anti*-coupling steps were modelled with isoquinoline as the substrate and compared to the ones computed for pyridine. However, despite numerous attempts no transition state leading to complex **5**, such as **^Iq^Anti-1-TS** depicted in Fig. 11, could be located. A scan of the potential energy surface along the C–C distance between the C1 and C1′ isoquinoline carbon atoms only showed shallow maxima (*cf.* ESI†). In this context, a comparison of the energies of the transition states obtained for the competition between *syn* and *anti*-CC couplings for pyridine and the value obtained for *syn*-CC coupling for isoquinoline are instructive (Fig. 11). Notably, the *anti*-coupling step for pyridine (**Anti-1-TS**) is 13.3 kcal mol^–1^ lower in energy than the corresponding *syn*-transformation (**Syn-1-TS**). With isoquinoline as the substrate, the Gibbs free energy for the *syn*-coupling transition state (**^Iq^Syn-1-TS**) was calculated to be 14 kcal mol^–1^ lower than that obtained for the pyridine transition state. Consequentially, if a similar energy lowering is applied to **^Iq^Anti-1-TS**, then the *anti*-CC coupling for isoquinoline should be associated with a very low activation barrier if any. Moreover, the resulting *anti*-CC coupling step is associated with a very exergonic transformation with Δ*G* = –19.7 kcal mol^–1^. It can be concluded that, in the case of isoquinoline, an *anti*-coupling is favoured over a *syn*-coupling, thus explaining the reactivity observed experimentally.

## Conclusions

We investigated the reductive coupling of pyridine mediated by the Zr^II^ synthon, ^Cbz^(PNP)ZrCl(η^6^-toluene) **1**. In this context, we were able to gain insights into the range of possible substrates and expanded the substrate scope for this reaction. Through experimental and extensive computational investigations, we were able to rule out several possible reaction paths and put forward a reaction mechanism which has been disregarded in previous publications concerning pyridine homocoupling reactions. To our knowledge, we report the first reductive coupling of pyridine proceeding *via* an initial *syn* C–C coupling step, instead of an initial C–H activation of the pyridine substrate. Further DFT calculations provided explanations for the observed deviation from the main reaction path in case of the substrates 2-picoline, DMAP and isoquinoline.

## Conflicts of interest

There are no conflicts to declare.

## Supplementary Material

Supplementary information

Supplementary information

Crystal structure data
