# Supplementary material for: Dehydrogenative coupling of 4-substituted pyridines mediated by a zirconium(ii) synthon: reaction pathways and dead ends
Source: Chem Sci. 2018 May 16;9(23):5223–32. doi: 10.1039/c8sc01025k (PMC6001252; doi:10.1039/c8sc01025k)
Supplement: Supplementary file 1 [file SC-009-C8SC01025K-s001.pdf]

Electronic Supplementary Material (ESI) for Chemical Science.
This journal is © The Royal Society of Chemistry 2018

Supporting Information

Dehydrogenative Coupling of 4-Substituted Pyridines Utilizing an Amidodiphosphine-Stabilized Zirconium(II) Synthon

Lukas S. Merz, Hubert Wadepohl, Eric Clot* and Lutz H. Gade*

Experimental Procedures S2
NMR Spectra S13
X-ray crystal Structure Determinations S27
UV/Vis Spectroscopy S31
DFT Calculations S32
References S35

Experimental Procedures

General Remarks

All manipulations were performed under an argon atmosphere using standard Schlenk and Glovebox techniques. Glassware was dried by heating to 150 °C overnight and evacuating during cooling down. The commercially available argon of purity 5.0 was further dried by passing over P_2O_5 granulate. Solvents were either dispensed from a MBRAUN-SPS-800 or in case of benzene dried over sodium/-benzophenone ketyl and stored over potassium mirrors. Air-sensitive compounds were stored and handled in a Glovebox Workstation (Unilab-200, MBRAUN). Chemicals were either procured from the Chemical Institute of the University of Heidelberg or purchased from Sigma-Aldrich, abcr or Acros. Pyridine substrates were dried using CaH_2 and degassed by three freeze-pump-thaw cycles prior to use. Deuterated solvents were purchased from Deutero or Sigma-Aldrich and dried over sodium.

^{1}H, ^{13}C and ^{31}P NMR spectra were recorded on a Bruker Avance III 600 or a Bruker II 400 spectrometer. Chemical shifts δ were measured relative to the shift of the residual protons in the deuterated solvent or the solvent resonances (benzene-d_6: δ = 7.16 ppm for ^{1}H, δ = 128.06 ppm for ^{13}C; toluene-*d8*: δ = 2.09 ppm for ^{1}H, δ = 20.04 ppm for ^{13}C) and are given in parts per million (ppm). ^{31}P spectra were referenced to external $P(OMe)_3$ (141.0 ppm with respect to 85 % H_3PO_4 at 0.0 ppm). Signals were assigned by analysis of two dimensional spectra (COSY, HSQC, HMBC). Coupling constants, ^{n}J, are stated in Hertz (Hz) and the signal patterns are denoted according to usual conventions (singlet = s, doublet = d, etc.). IN cases where an unambiguous assignment of aromatic signals was impossible due to overlapping or broadening, the designations Ar-H or Ar-C were employed. If not stated otherwise, all spectra were recorded at room temperature and proton decoupling algorithms were employed during the acquisition of ^{13}C as well as ^{31}P NMR spectra.

Microanalyses (C, H, N) were performed at the Department of Organic Chemistry at the University of Heidelberg on an Elementar vario MICRO Cube machine.

[^{iPr}(^{cbz}PNP)Zr(bipy-d_8)Cl] (**3-D**):

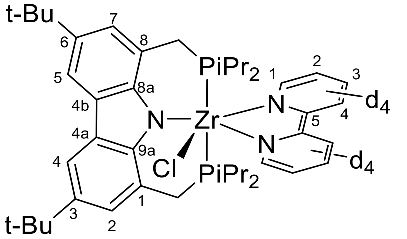

To a solution of [(Cbzdiphos^{iPr})Zr(tol)Cl] (100 mg, 133 µmol, 1.0 equiv) in toluene (10 ml), neat pyridine-d5 (40.6 µl, 277.2 µmol, 2.1 equiv) was added and heated to 50 °C for 19 h. Then, all volatiles were removed and the residue was dissolved in diethylether and filtrated over Celite®. Through slow evaporation of the solvent the titleproduct was received as a purple crystalline solid (23 mg, 28 µmol, 21 %).

^{1}H NMR (600.13 MHz, C_6D_6, 295 K): δ [ppm] = 8.21 (d, *J* = 2.1 Hz, 2H, $H_{Carb-4,5}$), 7.31 (d, *J* = 2.1 Hz, 2H, $H_{Carb-2,7}$), 3.50 (d, *J* = 14.3 Hz, 2H, C*H*$_2$), 3.43 – 3.33 (m, 2H, C*H*$_2$), 2.52 – 2.43 (m, 2H, C*H*(CH$_2$)$_2$), 2.35 (m, 2H, C*H*(CH$_3$)$_2$), 1.49 (s, 18H, C(C*H*$_3$)$_3$), 1.30 (dq, *J* = 15.6, 7.0 Hz, 12H, CH(C*H*$_3$)$_2$), 1.24 – 1.15 (m, 6H), 1.03 (q, *J* = 7.1 Hz, 6H, CH(C*H*$_3$)$_2$).

^{31}P-NMR (242.94 MHz, C_6D_6, 295 K): δ (ppm) = 21.5 (bs).

[^{iPr}(^{cbz}PNP)Zr($^{4\text{-}Me}$bipy)Cl] (**3-Me**):

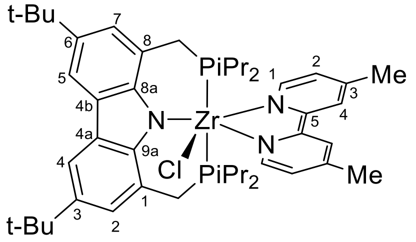

To a solution of [(Cbzdiphos^{iPr})Zr(tol)Cl] (100 mg, 133 µmol, 1.0 equiv.) in toluene (10 ml), 4-methylpyridine (28 mg, 279 µmol, 2.1 equiv) was added and heated to 60 °C for 24 h. Then, all volatiles were removed and the residue was recrystallized from hexane to yield a purple solid (20 mg, 23.6 µmol, 18 %).

^{1}H NMR (600.13 MHz, C_6D_6, 295 K): δ [ppm] = 9.18 (d, *J* = 7.1 Hz, 1H, H_{bipy-1}), 8.22 (d, *J* = 1.7 Hz, 2H, $H_{Carb-4,5}$), 7.34 (d, *J* = 1.7 Hz, 2H, $H_{Carb-2,7}$), 7.31 (bs, 1H, $H_{bipy-1'}$), 6.33 (s, 1H, $H_{bipy-4/4'}$), 6.31 (s, 1H, $H_{bipy-4/4'}$), 4.87 (d, *J* = 7.0 Hz, 1H, H_{bipy-2}), 4.29 (d, *J* = 7.0 Hz, 1H, $H_{bipy-2'}$), 3.56 (d, *J* = 14.3 Hz, 2H, C*H*$_2$), 3.41 (d, *J* = 14.3 Hz, 2H, C*H*$_2$), 2.57 – 2.50 (m, 2H, C*H*(CH$_3$)$_2$), 2.44 – 2.38 (m, 2H, C*H*(CH$_3$)$_2$), 1.86 (s, 3H, $H_{Bipy-Me}$), 1.76 (s, 3H, $H_{Bipy-Me}$), 1.51 (s, 18H, C(C*H*$_3$)$_3$), 1.38 (dd, *J* = 14.1, *J* = 7.0 Hz, 6H, CH(C*H*$_3$)$_2$), 1.33 (dd, *J* = 13.0, *J* = 7.0 Hz, 6H, CH(C*H*$_3$)$_2$), 1.29 (dd, *J* = 14.6, *J* = 7.0 Hz, 6H, CH(C*H*$_3$)$_2$), 1.07 (dd, *J* = 14.1, *J* = 7.1 Hz, 6H, CH(C*H*$_3$)$_2$).

^{13}C NMR (150.90 MHz, C_6D_6, 295 K): δ [ppm] = 144.95 (t, *J* = 3,3 Hz, C_{Carb}), 143.62 (s, $C_{bipy-1,1'}$), 141.74 (s, $C_{bipy-2,2'}$), 141.17 (s, C_{Carb}), 133.29 (s, C_{Carb}), 132.83 (s, $C_{bipy-3.3'}$), 129.33 (s, C_{Carb}), 126.21 (s, $C_{Carb-2,7}$), 125.70 (s, C_{Carb}), 120.81 (s), 120.05 (s, $C_{bipy-4,4'}$), 118.94 (s, $C_{bipy-4,4'}$), 114.75 (s, $C_{Carb-4,5}$), 111.94 (s, $C_{bipy-5,5'}$), 111.61 (s, C_{Carb}), 34.51 (s, C(*C*H$_3$)$_3$), 32.17 (s, *C*(CH$_3$)$_3$), 27.46 (d, *J* = 8.7 Hz, *C*H$_2$), 24.70 (t, *J* = 7.8 Hz, *C*H(CH$_2$)$_2$), 22.94 (t, *J* = 6.1 Hz, *C*H(CH$_2$)$_2$), 20.08 (s, $C_{Bipy-Me}$), 19.77 (s, $C_{Bipy-Me}$), 19.30 (s, CH(*C*H$_3$)$_2$), 19.06 (s, CH(*C*H$_3$)$_2$), 18.92 (t, *J* = 2.0 Hz, CH(*C*H$_3$)$_2$), 18.5 (s, CH(*C*H$_3$)$_2$).

^{31}P NMR (242.94 MHz, C_6D_6, 295 K): δ (ppm) = 23.4 (bs).

**Elemental analysis:**	calcd. for $C_{46}H_{66}N_3P_2ClZr$:	C 65.03 H 7.83 N 4.95 P 7.29 Cl 4.17 Zr 10.74.
	found:	C 65.10 H 7.99 N 4.73.

[(^{iPr}(^{cbz}PNP)Zr ($^{4\text{-}Et}$bipy)Cl] (**3-Et**):

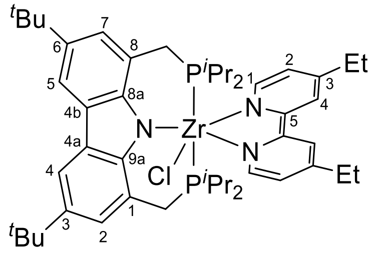

To a solution of [(Cbzdiphos^{iPr})Zr(tol)Cl] (250 mg, 330 µmol, 1.0 equiv) in benzene (20 ml), neat 4-ethylpyridine (79 µl, 693 µmol, 2.1 equiv) was added and the resulting mixture was heated to 50 °C for 19 h. Then, all volatiles were removed and the residue was dissolved in *n*-hexane, filtrated over Celite® and cooled to [illegible]40 °C. The titleproduct precipitated as a purple solid (70 mg, 82 µmol, 25 %).

^{1}H NMR (600.13 MHz, C_6D_6, 295 K): δ [ppm] = 9.19 (d, *J* = 7.1 Hz, 1H, $H_{Bipy\text{-}1}$), 8.23 (d, *J* = 2.0 Hz, 2H, $H_{Carb\text{-}4,5}$), 7.33 (d, *J* = 2.0 Hz, 2H, $H_{Carb\text{-}2,7}$), 7.28 (s, 1H, $H_{Bipy\text{-}1'}$), 6.45 (s, 1H, $H_{bipy\text{-}4/4'}$), 6.43 (s, 1H, $H_{bipy\text{-}4/4'}$), 4.91 (d, *J* = 7.1 Hz, 1H, $H_{bipy\text{-}2}$), 4.29 (d, *J* = 7.2 Hz, 1H, $H_{Bipy\text{-}2'}$), 3.54 (d, *J* = 14.4 Hz, 2H, CH_2), 3.42 (dt, *J* = 14.4, *J* = 3.0 Hz, 2H, CH_2), 2.55 – 2.48 (m, 2H, C*H*(CH_3)$_2$), 2.41 – 2.37 (m, 2H, , C*H*(CH_3)$_2$), 2.15 (q, *J* = 7.5 Hz, 2H, C$H_2$$CH_3$), 2.03 (q, *J* = 7.5 Hz, 2H, C$H_2$$CH_3$), 1.50 (s, 18H, C(C$H_3$)$_3$), 1.39 – 1.24 (m, 18H, CH(CH_3)$_2$), 1.06 (q, *J* = 7.1 Hz, 6H, CH(CH_3)$_2$), 0.97 (t, *J* = 7.5 Hz, 3H, CH_2CH_3), 0.80 (t, *J* = 7.5 Hz, 3H, CH_2CH_3).

^{13}C NMR (150.90 MHz, C_6D_6, 295 K): δ [ppm] = 144.97 (s, $C_{Carb\text{-}6}$), 143.79 (s, $C_{Bipy\text{-}1}$), 141.96 (s, $C_{Bipy\text{-}1'}$), 141.21 (s, $C_{Bipy\text{-}3,3'}$), 136.25 (s, C_{Carb}), 136.10 (s, C_{Carb}), 133.34 (s, $C_{Bipy\text{-}5/5'}$), 132.92 (s, $C_{Bipy\text{-}5/5'}$), 126.33 (t, *J* = 3.0 Hz, $C_{Carb\text{-}2,7}$), 120.81 (s, $C_{Carb\text{-}8}$), 118.47 (s, $C_{Bipy\text{-}4/4'}$), 117.45 (s, $C_{Bipy\text{-}4/4'}$), 114.72 (s, $C_{Carb\text{-}4,5}$), 110.74 (s, $C_{Bipy\text{-}2'}$), 110.48 (s, $C_{Bipy\text{-}2}$), 34.51 (s, *C*(CH_3)$_3$), 32.17 (s, C(*C*H_3)$_3$), 27.66 (s, Et-CH_2), 27.60 (t, *J* = 4.5 Hz, CH_2), 27.41 (s, Et-CH_2), 24.56 (t, *J* = 7.8 Hz, *C*H(CH_3)$_2$), 22.89 (t, *J* = 6.2 Hz, *C*H(CH_3)$_2$), 19.23 (s, CH(*C*H_3)$_2$), 19.09 (s, CH(*C*H_3)$_2$), 18.92 – 18.78 (m, CH(*C*H_3)$_2$), 18.59 (s, CH(*C*H_3)$_2$), 14.79 (s, Et-CH_3), 14.37 (s, Et-CH_3).

^{31}P-NMR (242.94 MHz, C_6D_6, 295 K): δ (ppm) = 22.1 (bs).

**Elemental analysis:**	calcd. for $C_{48}H_{70}N_3P_2ClZr$:	C 65.68 H 8.04 N 4.79 P 7.06 Cl 4.04 Zr 10.39.
	found:	C 65.37 H 8.24 N 5.23.

[(^{iPr}(^{cbz}PNP)Zr ($^{4\text{-tBu}}$bipy)Cl] (**3-tBu**):

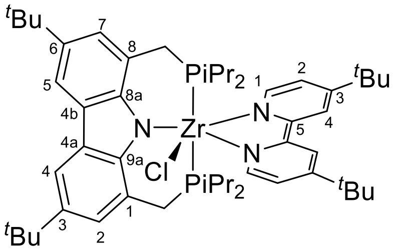

To a solution of [(Cbzdiphos^{iPr})Zr(tol)Cl] (50 mg, 66 µmol, 1.0 equiv) in benzene (1 ml), neat 4-tert-butylpyridine (10 mg, 132 µmol, 2.0 equiv) was added and heated to 50 °C for 19 h. Then, all volatiles were removed, the residue was washed with pentane/HMDSO (2:1, 1ml) and dried under vacuum to receive the product as a purple solid (21 mg, 22.5 µmol, 34 %).

^{1}H NMR (600.13 MHz, C_6D_6, 295 K): δ [ppm] = 9.22 (dd, *J* = 7.3, 0.8 Hz, 1H, $H_{bipy\text{-}1}$), 8.28 (d, *J* = 2.1 Hz, 2H, $H_{Carb\text{-}4,5}$), 7.34 (d, *J* = 2.1 Hz, 2H, $H_{Carb\text{-}2,7}$), 7.18 (s, 1H, $H_{bipy\text{-}1'}$), 6.92 – 6.66 (m, 2H, $H_{bipy\text{-}4,4'}$), 5.30 – 5.02 (m, 1H, $H_{bipy\text{-}2}$), 4.36 (d, *J* = 7.2 Hz, 1H, $H_{bipy\text{-}2'}$), 3.53 (d, *J* = 14.5 Hz, 2H, C*H*$_2$), 3.51 – 3.41 (m, 2H, C*H*$_2$), 2.55 – 2.44 (m, 2H, C*H*(CH_3)$_2$), 2.42 – 2.32 (m, 2H, C*H*(CH_3)$_2$), 1.51 (s, 18H, C(C*H*$_3$)$_3$), 1.36 (q, *J* = 7.0 Hz, 6H, CH(C*H*$_3$)$_2$), 1.31 (q, *J* = 7.0 Hz, 6H, CH(C*H*$_3$)$_2$), 1.23 (q, *J* = 7.3 Hz, 6H, CH(C*H*$_3$)$_2$), 1.11 (s, 9H, $H_{Bipy\text{-}tBu}$), 1.07 (q, *J* = 7.3 Hz, 6H, CH(C*H*$_3$)$_2$), 0.92 (s, 9H, $H_{Bipy\text{-}tBu}$).

^{13}C NMR (150.90 MHz, C_6D_6, 295 K): δ [ppm] = 145.4 (s, $C_{Carb\text{-}8a,9a}$), 143.8 (s, $C_{bipy\text{-}1}$), 142.8 (s, $C_{bipy\text{-}3}$), 142.7 (s, $C_{bipy\text{-}3,3'}$) 142.0 (s, $C_{bipy\text{-}1'}$), 141.4 (s, $C_{Carb\text{-}3,6}$), 133.5 (s, $C_{bipy\text{-}5}$), 133.6 (s, $C_{bipy\text{-}5'}$), 126.6 (t, *J* = 3.1 Hz, $C_{Carb\text{-}2,7}$), 120.8 (s, $C_{Carb\text{-}1,8}$), 115.1 (s, $C_{bipy\text{-}4,4'}$), 114.7 (s, $C_{Carb\text{-}4,5}$), 114.1 (s, $C_{Carb\text{-}4a,4b}$), 108.5 (s, $C_{bipy\text{-}2}$), 108.2 (s, $C_{bipy\text{-}2'}$), 34.5 (s, *C*(CH_3)$_3$), 33.2 (s, $C_{Bipy\text{-}CMe3}$), 33.0 (s, $C_{Bipy\text{-}CMe3}$), 32.2 (s, C(*C*H$_3$)$_3$), 30.0 (s, $C_{Bipy\text{-}tBu}$), 29.9 (s, $C_{Bipy\text{-}tBu}$) 27.8 (t, *J* = 4.6 Hz, *C*H$_2$), 24.4 (t, *J* = 7.8 Hz, *C*H(CH_3)$_2$), 22.8 (t, *J* = 6.2 Hz, *C*H(CH_3)$_2$), 19.2 (s, CH(*C*H$_3$)$_2$), 19.1 (s, CH(*C*H$_3$)$_2$), 18.8 (s, CH(*C*H$_3$)$_2$), 18.6 (s, CH(*C*H$_3$)$_2$).

^{31}P-NMR (242.94 MHz, C_6D_6, 295 K): δ (ppm) = 21.5 (bs).

**Elemental analysis:**	calcd. for $C_{46}H_{66}N_3P_2ClZr \cdot 0.5HMDSO$:	C 65.08 H 8.64 N 4.14 P 6.10, Cl 3.49 O 0.79 Si 2.77 Zr 8.99
	found:	C 65.29 H 8.36 N 4.35

[(^{iPr}(^{cbz}PNP)Zr ($^{4-Bn}$bipy)Cl] (**3-Bn**):

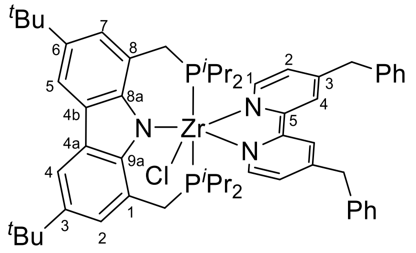

To a solution of [(Cbzdiphos^{iPr})Zr(tol)Cl] (50 mg, 66 µmol, 1.0 equiv) in benzene (1 ml), neat 4-benzylpyridine (xx mg, xx µmol, 2.0 equiv) was added and heated to 50 °C for 19 h. Then, all volatiles were removed, and the residue was extracted with pentane/HMDSO (2:1, 1ml). After removal of the solvent the product was as a purple solid (28 mg, 28 µmol, 42 %).

^{1}H NMR (600.13 MHz, C_6D_6, 295 K): δ [ppm] = 9.15 (d, *J* = 7.1 Hz, 1H, H_{Bipy-1}), 8.23 (d, *J* = 2.0 Hz, 2H, $H_{Carb-4,5}$), 7.35 (s, 1H, $H_{Bipy-1'}$), 7.31 (d, *J* = 2.0 Hz, 2H, $H_{Carb-2,7}$), 7.22 (dd, *J* = 8.3, 7.0 Hz, 2H, H_{Ph}), 7.14 – 7.08 (m, 5H, H_{Ph}), 7.05 – 6.99 (m, 1H, H_{Ph}), 6.95 – 6.92 (m, 2H, H_{Ph}), 6.47 (s, 1H, $H_{Bipy-4/4'}$), 6.44 (s, 1H, $H_{Bipy-4/4'}$), 4.85 (d, *J* = 7.1 Hz, 1H, H_{Bipy-2}), 4.26 (d, *J* = 7.2 Hz, 1H, $H_{Bipy-2'}$), 3.50 (d, *J* = 14.4 Hz, 2H, CH_2), 3.42 (s, 2H, CH_2Ph), 3.37 (dt, *J* = 14.4, 3.1 Hz, 2H, CH_2), 3.29 (s, 2H, CH_2Ph), 2.55 – 2.42 (m, 2H, C*H*(CH_3)$_2$), 2.41 – 2.28 (m, 2H, , C*H*(CH_3)$_2$), 1.49 (s, 18H, C(C*H*$_3$)$_3$), 1.34 (q, *J* = 7.0 Hz, 6H, CH(C*H*$_3$)$_2$), 1.27 (dq, *J* = 21.6 Hz, *J* = 7.0 Hz, 12H, CH(C*H*$_3$)$_2$), 1.04 (q, *J* = 7.0 Hz, 6H, CH(C*H*$_3$)$_2$).

^{13}C NMR (150.90 MHz, C_6D_6, 295 K): δ [ppm] = 144.98 (t, *J* = 3.3 Hz, $C_{Carb-8a,9a}$), 144.01 (s, C_{Bipy-1}), 142.23 (s, $C_{Bipy-1'}$), 141.58 (s, $C_{Bipy-Ph}$), 141.46 (s, $C_{Bipy-Ph}$), 141.27 (s, $C_{Carb-3,6}$), 133.32 (s, $C_{Bipy-3'}$), 132.96 (d, *J* = 1.8 Hz, C_{Bipy-3}), 128.15 (s, $C_{Bipy-Ph}$), 129.08 (s, $C_{Bipy-Ph}$), 128.63 (s, $C_{Bipy-Ph}$), 128.59 (s, $C_{Bipy-Ph}$), 128.06 (partially overlapping with benzene-d6, $C_{Carb-4a,b}$), 126.45 – 126.16 (m, $C_{Carb-2,7}$, $C_{Bipy-Ph}$), 120.67 (s, $C_{Carb-1,8}$), 120.42 (s, $C_{Bipy-4/4'}$), 11 9.21 (s, $C_{Bipy-4/4'}$), 114.70 (s, $C_{Carb-4,5}$), 111.12 (s, $C_{Bipy-2'}$), 110.94 (s, C_{Bipy-2}), 40.79 (s, *C*H_2Ph), 40.67 (s, *C*H_2Ph), 34.50 (s, *C*(CH_3)$_3$), 32.16 (s, C(*C*H_3)$_3$), 27.67 (t, *J* = 4.6 Hz, *C*H_2), 24.66 (t, *J* = 7.8 Hz, *C*H(CH_3)$_2$), 22.85 (t, *J* = 6.2 Hz, *C*H(CH_3)$_2$), 19.28 (s, CH(*C*H_3)$_2$), 18.96 (s, CH(*C*H_3)$_2$), 18.85 (s, CH(*C*H_3)$_2$), 18.54 (s, CH(*C*H_3)$_2$).

^{31}P-NMR (242.94 MHz, C_6D_6, 295 K): δ (ppm) = 20.4 (bs).

**Elemental analysis:**	calcd. for $C_{48}H_{70}N_3P_2ClZr$·0.5HMDSO:	C 67.65 H 7.72 N 3.88 P 5.72 Cl 3.27 Si 2.59 O 0.74 Zr 8.42.
	found:	C 67.25 H 7.25 N 4.00.

[(^{iPr}(^{cbz}PNP)Zr ($^{4\text{-}Ph}$bipy)Cl] (**3-Ph**):

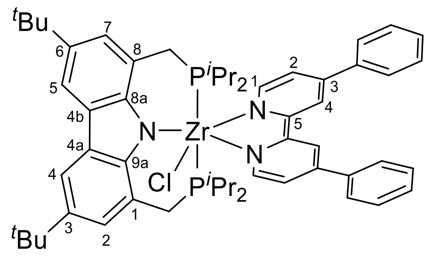

To a solution of [(Cbzdiphos^{iPr})Zr(tol)Cl] (393 mg, 522 µmol, 1.0 equiv) in toluene (20 ml), neat 4-phenylpyridine (170 µl, 1.1 mmol, 2.1 equiv) was added and the resulting mixture was heated to 60 °C for 19 h. Then the solvent was removed, the residue was washed with hexane (4 ml) and dried under vacuum. The titleproduct was received as a deep blue solid (300 mg, 308 µmol. 59 %).

^{1}H NMR (600.13 MHz, C_6D_6, 295 K): δ [ppm] = 9.33 (d, *J* = 7.2 Hz, 1H, $H_{Bipy\text{-}1}$), 8.30 (d, *J* = 2.1 Hz, 2H, $H_{Carb\text{-}4,5}$), 7.41 – 7.39 (m, 2H, $H_{Bipy\text{-}Ph}$), 7.36 (d, *J* = 2.1 Hz, 2H, $H_{Carb\text{-}2,7}$), 7.34 (s, 1H, $H_{Bipy\text{-}4}$), 7.31 (d, *J* = 1.9 Hz, 1H, $H_{Bipy\text{-}4'}$), 7.27 (d, *J* = 7.2 Hz, 1H, $H_{Bipy\text{-}Ph}$), 7.21 (bs, 1H, $H_{Bipy\text{-}1'}$) 7.14 – 7.10 (m, 3H, $H_{Bipy\text{-}Ph}$), 7.06 (t, *J* = 7.8 Hz, 2H, $H_{Bipy\text{-}Ph}$), 7.00 (t, *J* = 7.6 Hz, 1H, $H_{Bipy\text{-}Ph}$), 6.91 (t, *J* = 7.3 Hz, 1H, $H_{Bipy\text{-}Ph}$), 5.64 (dt, *J* = 7.2, 1.3 Hz, 1H, $H_{Bipy\text{-}2}$), 4.95 – 4.90 (m, 1H, $H_{Bipy\text{-}2'}$), 3.57 – 3.47 (m, 4H, $C\mathit{H}_2$), 2.48 – 2.40 (m, 2H, $C\mathit{H}(CH_3)_2$), 2.40 – 2.33 (m, 2H, $C\mathit{H}(CH_3)_2$), 1.52 (s, 18H, $C(C\mathit{H}_3)_3$), 1.31 (m, 12H, $CH(C\mathit{H}_3)_2$), 1.18 (q, *J* = 7.2 Hz, 6H, $CH(C\mathit{H}_3)_2$), 1.08 (q, *J* = 7.2 Hz, 6H, $CH(C\mathit{H}_3)_2$).

^{13}C NMR (150.90 MHz, C_6D_6, 295 K): δ [ppm] = 145.12 (t, *J* = 3.2 Hz, $C_{Carb\text{-}8a,9a}$), 144.62 (s, $C_{Bipy\text{-}1}$), 142.28 (s, $C_{Bipy\text{-}1'}$), 141.77 (s, $C_{Carb\text{-}3,6}$), 138.12 (s, $C_{Bipy\text{-}3}$), 137.66 (s, $C_{Bipy\text{-}3'}$), 135.62 (s, $C_{Bipy\text{-}5/5'}$), 135.46 (s, $C_{Bipy\text{-}5/5'}$), 131.95 (s, $C_{Bipy\text{-}Ph}$), 131.84 (s, $C_{Bipy\text{-}Ph}$), 129.19 (s, $C_{Bipy\text{-}Ph}$), 128.79 (s, $C_{Bipy\text{-}Ph}$), 128.59 (s, $C_{Bipy\text{-}Ph}$), 128.06 (partially overlapping with benzene-d6, $C_{Carb\text{-}4a,b}$), 127.24 (s, $C_{Bipy\text{-}Ph}$), 127.01 (m, $C_{Bipy\text{-}Ph}$), 126.61 (t, *J* = 3.1 Hz, $C_{Carb\text{-}2,7}$), 125.04 (s, $C_{Bipy\text{-}Ph}$), 124.96 (s, $C_{Bipy\text{-}Ph}$), 121.59 (s, $C_{Bipy\text{-}Ph}$), 120.88 (s, $C_{Carb\text{-}1,8}$), 117.84 (s, $C_{Bipy\text{-}4}$), 116.76 (s, $C_{Bipy\text{-}4'}$), 114.85 (s, $C_{Carb\text{-}4,5}$), 108.62 (s, $C_{Bipy\text{-}2'}$), 108.14 (s, $C_{Bipy\text{-}2}$), 34.55 (s, $\mathit{C}(CH_3)_3$), 32.15 (s, $C(\mathit{C}H_3)_3$), 27.95 (t, *J* = 4.9 Hz, $\mathit{C}H_2$), 24.47 (t, *J* = 7.7 Hz, $\mathit{C}H(CH_3)_2$), 22.96 (t, *J* = 6.3 Hz, $\mathit{C}H(CH_3)_2$), 19.24 (s, $CH(\mathit{C}H_3)_2$), 19.00 (s, $CH(\mathit{C}H_3)_2$), 18.75 (t, *J* = 2.1 Hz, $CH(\mathit{C}H_3)_2$), 18.60 (s, $CH(\mathit{C}H_3)_2$).

^{31}P-NMR (242.94 MHz, C_6D_6, 295 K): δ (ppm) = 18.9 (bs).

**Elemental analysis:**	calcd. for $C_{56}H_{70}N_3P_2ClZr$:	C 69.07 H 7.25 N 4.32 P 6.36 Cl 3.64 Zr 9.37
	found:	C 68.32 H 7.52 N 4.38<br>Elemental analysis data repeatedly showed low carbon values, however, a good agreement with calculated hydrogen and nitrogen values. We attribute this to an incomplete combustion resulting in the formation of a zirconium-carbide species.

[(^{iPr}(^{cbz}PNP)Zr ($^{4\text{-}Sty}$bipy)Cl] (**3-Sty**):

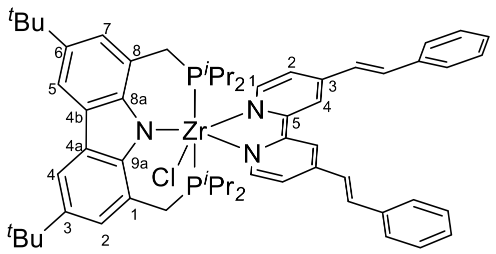

To a solution of [(Cbzdiphos^{iPr})Zr(tol)Cl] (50 mg, 66 µmol, 1.0 equiv) in benzene (1 ml), neat 4-styrylpyridine (22.7 mg, 125 µmol, 1.9 equiv) was added and heated to 50 °C for 19 h. Then, pentane (1 ml) was added, the reaction mixture was filtered through a syringe filter and cooled to 0 °C. The titleproduct precipitated as a green solid (30 mg, 29 µmol, 44 %).

^{1}H NMR (600.13 MHz, C_6D_6, 295 K): δ [ppm] = 9.18 (d, *J* = 7.2 Hz, 1H, H_{Bipy-1}), 8.31 (d, *J* = 2.1 Hz, 2H, $H_{Carb-4,5}$), 7.37 – 7.35 (m, 3H, $H_{Bipy-Ph}$), 7.35 (d, *J* = 2.1 Hz, 2H, $H_{Carb-2,7}$), 7.30 – 7.25 (m, 2H, $H_{Bipy-Ph}$), 7.19 (t, *J* = 7.8 Hz, 2H, $H_{Bipy-Ph}$), 7.14 (d, *J* = 8.1 Hz, 2H, $H_{Bipy-Ph}$), 7.02 – 6.99 (m, 1H, $H_{Bipy-Ph}$), 6.98 – 6.94 (m, 1H, $H_{Bipy-2'}$), 6.78 – 6.75 (m, 2H, CHC*H,* H_{Bipy-4}), 6.72 – 6.67 (m, 2H, CHC*H*, $H_{Bipy-4'}$), 6.63 (d, *J* = 16.0 Hz, 1H, CHC*H*), 6.26 (d, *J* = 15.9 Hz, 1H, CHC*H*), 5.62 – 5.51 (m, 1H, H_{Bipy-2}), 4.82 (dd, *J* = 7.4, 1.7 Hz, 1H, $H_{Bipy-2'}$), 3.54 (d, *J* = 14.3 Hz, 2H, C*H*$_2$), 3.44 (d, *J* = 14.3 Hz, 2H, C*H*$_2$), 2.35 (dq, *J* = 12.2, 5.8, 4.5 Hz, 4H, C*H*$(CH_3)_2$), 1.51 (s, 18H, C(C*H*$_3$)$_3$), 1.34 – 1.22 (m, 12H, CH(C*H*$_3$)$_2$), 1.14 (dd, *J* = 9.1, 5.7 Hz, 6H, CH(C*H*$_3$)$_2$), 1.07 (q, *J* = 7.2 Hz, 6H, CH(C*H*$_3$)$_2$).

^{13}C NMR (150.90 MHz, C_6D_6, 295 K): δ [ppm] = 145.09 (t, *J* = 3.2 Hz, $C_{Carb-8a,9a}$), 143.98 (s, C_{Bipy-1}), 142.00 (s, $C_{Carb-3,6}$), 141.35 (s, $C_{Bipy-1'}$), 138.17 (s, $C_{Bipy-Ph}$), 137.95 (s, $C_{Bipy-Ph}$), 136.01 (s, $C_{Bipy-5/5'}$), 135.90 (s, $C_{Bipy-5/5'}$), 130.66 (s, $C_{Bipy-3/3'}$), 130.64 (s, $C_{Bipy-3/3'}$), 128.85 (s, $C_{Bipy-Ph}$), 128.75 (s, $C_{Bipy-Ph}$), 127.21 (m, $C_{Bipy-Ph}$), 126.70 (d, *J* = 3.4 Hz, $C_{Carb-2,7}$), 126.27 (s, CH*C*H), 126.16 (s, CH*C*H), 125.78 (s, CH*C*H), 125.62 (s, CH*C*H), 121.25 (s, C_{Bipy-4}), 120.89 (s, $C_{Carb-1,8}$), 120.29 (s, $C_{Bipy-4'}$), 114.89 (s, $C_{Carb-4,5}$), 106.43 (s, $C_{Bipy-2'}$), 106.01 (s, C_{Bipy-2}), 34.56 (s, *C*$(CH_3)_3$), 32.13 (s, C(*C*H$_3$)$_3$), 27.95 (t, J = 4.7 Hz, CH_2), 24.36 (t, *J* = 7.7 Hz, *C*H$(CH_3)_2$), 22.98 (t, *J* = 6.5 Hz, *C*H$(CH_3)_2$), 19.26 (s, CH(*C*H$_3$)$_{2z}$), 18.86 (s, CH(*C*H$_3$)$_2$), 18.68 (t, *J* = 2.1 Hz, CH(*C*H$_3$)$_2$), 18.61 (s, CH(*C*H$_3$)$_2$).

^{31}P-NMR (242.94 MHz, C_6D_6, 295 K): δ (ppm) = 17.6 (bs).

**Elemental analysis:**	calcd. for $C_{60}H_{74}N_3P_2ClZr$:	C 70.25 H 7.25 N 4.10 Cl 3.46 P 6.04 Zr 8.89
	found:	C 69.45 H 6.90 N 4.14<br>Elemental analysis data repeatedly showed low carbon values, however, a good agreement with calculated hydrogen and nitrogen values. We attribute this to an incomplete combustion resulting in the formation of a zirconium-carbide species.

[(^{iPr}(^{cbz}PNP)Zr (µ^2-DMAP)(dmap)Cl] (**4**):

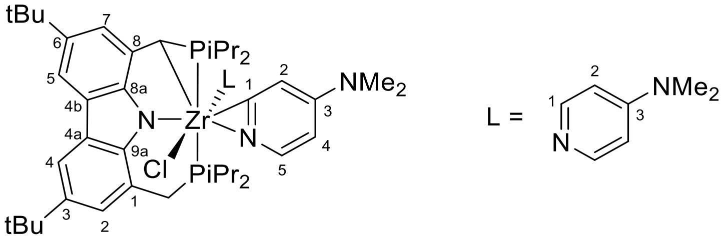

To a solution of [(Cbzdiphos^{iPr})Zr(tol)Cl] (50 mg, 66 µmol, 1.0 equiv) in benzene (1 ml), a solution of DMAP (16 mg, 132 µmol, 2.0 equiv) in benzene (1 ml) was added and the mixture was stirred at room temperature for 20 h. To this solution hexane (2 ml) was added and the reaction mixture was cooled until a precipitate had formed. After filtration the product was obtained as a light brown solid (25 mg, 28 µmol, 42 %).

^{1}H NMR (600.13 MHz, C_6D_6, 295 K): δ [ppm] = 8.47 (s, *J* = 6.1 Hz, 1H, H_{DMAP-5}), 8.33 (s, 1H, H_{Carb-4}), 8.26 (s, 2H, $H_{dmap-1,1'}$), 7.87 (s, 1H, H_{Carb-5}), 7.59 (s, 1H, H_{Carb-2}), 7.33 (s, 1H, H_{DMAP-2}), 6.94 (s, 1H, H_{Carb-7}), 6.26 (dd, *J* = 6.1 Hz, *J* = 2.4 Hz, 1H, H_{DMAP-4}), 5.68 (s, 2H, $H_{dmap-2,2'}$), 4.95 (dd, *J* = 16.0, *J* = 4.4 Hz, 1H, C*H*2), 3.60 (dd, *J* = 14.9 Hz, 1H, C*H*), 2.87 (dt, *J* = 14.2 Hz, *J* = 7.3 Hz, 1H, C*H*(CH_3)$_2$), 2.48 (dtt, *J* = 10.1 Hz, *J* = 7.3, *J* = 2.6 Hz, 1H, C*H*(CH_3)$_2$), 2.39 (m, 7H, CH, $NMe_{2\text{-}DMAP}$), 2.08 (bs, 6H, $NMe_{2\text{-}dmap}$), 1.78 (ddd, *J* = 19.4 Hz, *J* = 12.0 Hz, *J* = 7.2 Hz, 6H, CH(C*H*3)$_2$), 1.61 (s, 9H, C(C*H*3)$_3$), 1.55 – 1.49 (m, 3H, CH(C*H*3)$_2$), 1.48 (s, 9H, C(C*H*3)$_2$), 1.39 (dd, *J* = 9.8, 7.1 Hz, 3H, CH(C*H*3)$_2$), 1.14 (dd, *J* = 11.0 Hz, *J* = 7.2 Hz, 3H, CH(C*H*3)$_2$), 0.88 – 0.75 (m, 7H, CH(C*H*3)$_2$, C*H*(CH_3)$_2$), 0.67 (dd, *J* = 15.1 Hz, *J* = 7.3 Hz, 3H, CH(C*H*3)$_2$), 0.38 (q, *J* = 7.1 Hz, 1H, C*H*(CH_3)$_2$).

^{13}C NMR (150.90 MHz, C_6D_6, 295 K): δ [ppm] = 209.38 – 209.07 (m, C_{DMAP-1}), 153.55 (s, C_{dmap-3}), 153.42 (s, C_{DMAP-3}), 150.53 (bs, $C_{dmap-1,1'f}$), 150.36 (d, *J* = 4.9 Hz, C_{Carb}), 146.01 (d, *J* = 2.7 Hz, C_{Carb-1}), 141.66 (s, C_{DMAP-5}), 140.08 (d, *J* = 2.3 Hz, $C_{Carb-3/6}$), 139.67 (s, $C_{Carb-3/6}$), 133.34 (d, *J* = 3.2 Hz, C_{Carb-8}), 127.21 (s, C_{Carb}), 122.84 (d, *J* = 6.8 Hz, C_{Carb-2}), 122.25 (s, $C_{Carb-9a}$), 121.78 (d, *J* = 2.1 Hz, C_{Carb}), 120.17 (s, C_{Carb-7}), 115.00 (s, C_{Carb-4}), 111.03 (s, C_{DMAP-2}), 109.27 (s, C_{DMAP-4}), 108.78 (s, C_{Carb-5}), 105.80 (s, $C_{dmap-2,2'}$), 46.65 (d, *J* = 53.6 Hz, *C*H), 38.83 (s, $NMe_{2\text{-}DMAP}$), 38.20 (s, $NMe_{2\text{-}dmap}$), 34.9 (s, *C*(CH_3)$_3$), 34.84 (s, *C*(CH_3)$_3$), 32,74 (s, C(*C*H3)$_3$), 32.69 (s, C(*C*H3)$_3$), 28.94 (d, *J* = 6.6 Hz, *C*H2), 26.45 – 26.28 (m, *C*H(CH_2)$_2$), 26.19 (s, *C*H(CH_2)$_2$), 25.74 (s, *C*H(CH_2)$_2$), 24.47 (d, *J* = 6.6 Hz, *C*H(CH_2)$_2$), 21.85 (d, *J* = 2.8 Hz, CH(*C*H3)$_2$), 21.52 (d, *J* = 9.8 Hz, CH(*C*H3)$_2$), 21.25 (d, *J* = 7.5 Hz, CH(*C*H3)$_2$), 21.07 – 20.93 (m, CH(*C*H3)$_2$), 20.79 (d, *J* = 6.5 Hz, CH(*C*H3)$_2$), 20.46 (d, *J* = 6.7 Hz, CH(*C*H3)$_2$), 19.01 (d, *J* = 4.0 Hz, CH(*C*H3)$_2$).

^{31}P-NMR (242.94 MHz, C_6D_6, 295 K): δ (ppm) = 7.2 (d, *J* = 22.1 Hz), 2.1 (d, *J* = 22.1 Hz)

**Elemental analysis:**	calcd. for $C_{48}H_{72}N_5P_2ClZr$:	C 63.51, H 8.00, N 7.72, P 6.82, Cl 3.91 Zr 10.05.
	found:	C 63.73, H 8.09, N 7.68.

[(^{iPr}(^{cbz}PNP)Zr (bisisoquinoline)Cl] (**5**):

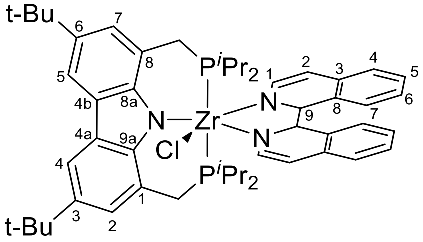

To a solution of [(Cbzdiphos^{iPr})Zr(tol)Cl] (50 mg, 66 µmol, 1.0 equiv) in benzene (1 ml), isoquinoline (17 mg, 132 µmol, 2.0 equiv) was added and heated to 50 °C for 12 h. Then, all volatiles were removed and the residue was washed with pentane/HMDSO (2:1, 2ml) to yield a red-brown solid (26 mg, 28 µmol, 43 %).

^{1}H NMR (600.13 MHz, C_6D_6, 295 K): δ [ppm] = 8.90 (d, *J* = 7.2 Hz, 1H, $H_{Isoquin-1}$), 8.34 (dd, *J* = 8.2, 1.4 Hz, 2H, H_{Carb-2}), 7.25 (s, 1H, H_{Carb-4}), 7.19 (s, 1H, $H_{Carb-4'}$), 7.09 (t, *J* = 7.4 Hz, 1H, $H_{Isoquin-5'}$), 6.99 (dd, *J* = 7.6, 1.0 Hz, 1H, $H_{Isoquin-4'}$), 6.94 – 6.90 (m, 1H, $H_{Isoquin-5}$), 6.84 (td, *J* = 7.5, 1.1 Hz, 1H, $H_{Isoquin-6'}$), 6.80 – 6.75 (m, 3H, $H_{Isoquin-6,7,7'}$), 6.59 (d, *J* = 7.2 Hz, 1H, $H_{Isoquin-4}$), 6.39 (d, *J* = 7.2 Hz, 1H, $H_{Isoquin-2'}$), 5.88 (d, *J* = 8.0 Hz, 1H, $H_{Isoquin-9}$), 5.81 (d, *J* = 7.3 Hz, 1H, $H_{Isoquin-2}$), 5.72 (d, *J* = 7.8 Hz, 1H, $H_{Isoquin-9'}$), 4.92 (d, *J* = 7.2 Hz, 1H, $H_{Isoquin-1'}$), 3.86 (dd, *J* = 14.4, 4.3 Hz, 1H, C*H*$_2$), 3.79 (dd, *J* = 14.4, 4.7 Hz, 1H, C*H*$_2$), 3.25 (td, *J* = 15.3, 6.4 Hz, 2H, C*H*$_2$), 2.16 (dq, *J* = 14.2, 7.1 Hz, 1H, C*H*Me$_2$), 2.06 (dq, *J* = 14.4, 7.1 Hz, 1H, C*H*Me$_2$), 1.80 (dq, *J* = 14.0, 7.0 Hz, 1H, C*H*Me$_2$), 1.73 (dt, *J* = 14.3, 7.0 Hz, 1H, C*H*(CH_3)$_2$), 1.50 (s, 9H, C(CH_3)$_3$), 1.47 (s, 9H, C(CH_3)$_3$), 1.24 (td, *J* = 13.4 Hz, *J* = 7.3 Hz, 3H, CH(C*H*$_3$)$_2$), 1.07 (dd, *J* = 13.3, 7.2 Hz, 3H, CH(C*H*$_3$)$_2$), 0.99 (dd, *J* = 13.5, 7.1 Hz, 3H, CH(C*H*$_3$)$_2$), 0.85 – 0.81 (m, 9H, CH(C*H*$_3$)$_2$), 0.81 – 0.77 (m, 3H, CH(C*H*$_3$)$_2$), 0.71 (dd, *J* = 14.4, 7.3 Hz, 3H, CH(C*H*$_3$)$_2$).

^{13}C NMR (150.90 MHz, C_6D_6, 295 K): δ [ppm] = 147.3 (dd, *J* = 4.1, 1.2 Hz, $C_{Carb-8a,9a}$), 146.9 (dd, *J* = 4.3, 1.0 Hz, $C_{Carb-3/6'}$), 146,7 (s, $C_{Isoquin-1}$), 141.9 (d, *J* = 4.0 Hz, $C_{Carb-3/6'}$), 141.8 (s, $C_{Isoquin-2'}$), 135.4 (s, $C_{Isoquin-3}$), 134.8 (s, $C_{Isoquin-3'}$), 133.5 (s, $C_{Isoquin-8'}$), 132.9 (s, $C_{Isoquin-8}$), 128.6 (d, *J* = 1.3 Hz, $C_{Carb-1/8}$), 128.4 (d, *J* = 1.4 Hz, $C_{Carb-1/8}$), 127.4 (d, *J* = 21.0 Hz $C_{Isoquin-5,5'}$), 126.7 (dd, *J* = 11.0, 6.2 Hz $C_{Carb-2,7'}$), 125.4 (s, $C_{Isoquin-6'}$), 125.4 (s, $C_{Isoquin-6}$), 125.0 (s, $C_{Isoquin-7/7'}$), 125.0 (s, $C_{Isoquin-7/7'}$), 122.6 (s, $C_{Isoquin-4'}$), 122.5 (s, $C_{Isoquin-4}$), 121.3 (s, $C_{Carb-4a/4b}$), 121.0 (s, $C_{Carb-4a/4b}$), 114.9 (d, *J* = 16.2 Hz, $C_{Carb-4,5}$), 104.4 (s, $C_{Isoquin-2}$), 104.0 (s, $C_{Isoquin-1'}$), 68.2 (s, $C_{Isoquin-9}$), 67.4 (s, $C_{Isoquin-9'}$), 34.5 (s, *C*(CH_3)$_3$), 32.1 (s, C(*C*H_3)$_3$), 29.2 (dd, *J* = 48.1, 10.4 Hz, CH_2), 23.7 (m, *C*HMe$_2$), 23.3 (dd , *J* = 12.5, 0.8 Hz, *C*HMe$_2$), 19.5 (s, CH(*C*H_3)$_2$), 19.2 (d, *J* = 3.2 Hz, CH(*C*H_3)$_2$), 19.2 (s, CH(*C*H_3)$_2$), 19.0 (s, CH(*C*H_3)$_2$), 18.8 (s, CH(*C*H_3)$_2$), 18.5 (s, CH(*C*H_3)$_2$), 18.2 (s, CH(*C*H_3)$_2$).

^{31}P-NMR (242.94 MHz, C_6D_6, 295 K): δ (ppm) = 7.41 – 1.22 (m)

**Elemental analysis:**	calcd. for C52$H_{68}N_3P_2$ClZr:	C 67.61 H 7.42 N 4.55 P 6.71 Cl 3.84 Zr 9.88.
	found:	C 67.98 H 7.27 N 4.36.

Determination of the KIE:

A solution of [(Cbzdiphos^{iPr})Zr(tol)Cl] (20.6 mg, 26.4 µmol, 1.00 equiv), pyridine (5.89 mg, 74.46 µmol, 2.82 equiv) and pyridine-d_5 (5.91 mg, 70.25 µmol, 2.66 equiv) in benzene-d_6 (0.5 ml) was heated to 50 °C for 8 h. The degree of conversion of pyridine vs. pyridine-d_5 was determined through the comparison of the relative intensity of H_{Bipy-1} compared to the CH_2 bridges of the ligand backbone.

$$KIE = \frac{k_1}{k_2} = \frac{\ln(1-F_1)}{\ln(1-F_2)}$$

F_1 and F_2 refer to the fraction of the conversions of the isotopic species (F_1:pyridine-d_5; F2: pyridine)

NMR Spectra

$[^{iPr}(^{cbz}PNP)Zr(bipy\text{-}d_8)Cl]$ (3-D):

^{1}H NMR (600.13 MHz, C_6D_6, 295 K):

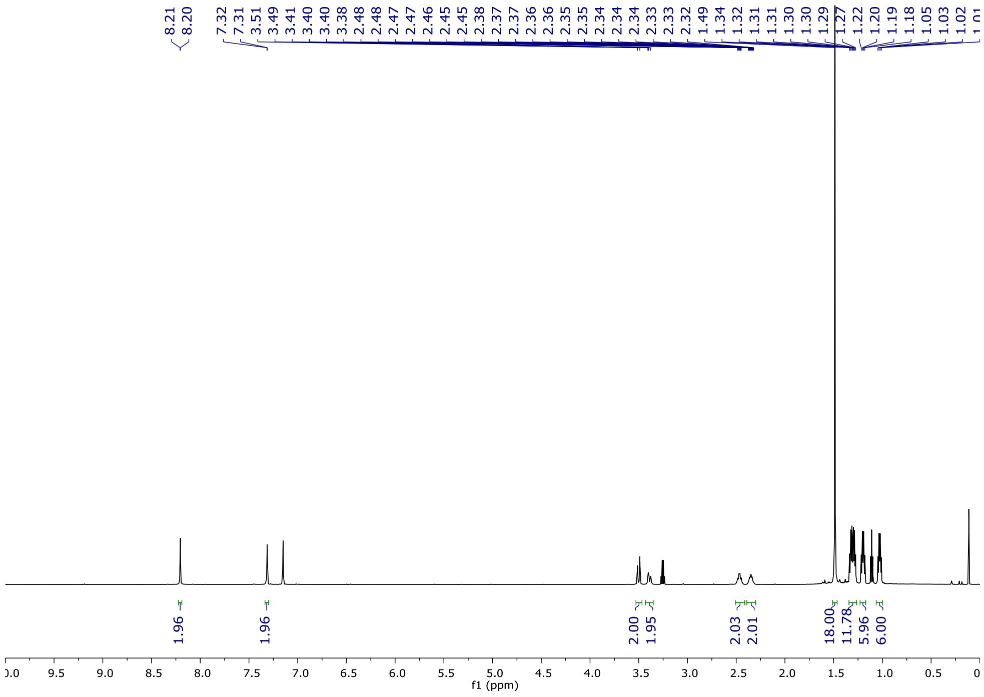

^{13}C NMR (150.90 MHz, C_6D_6, 295 K):

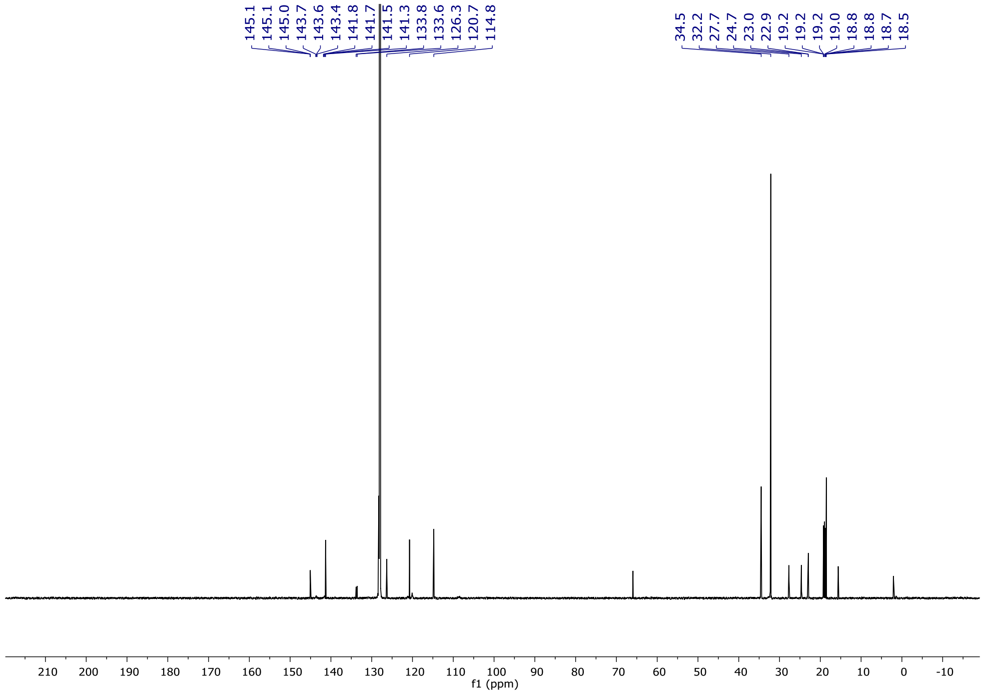

^{31}P-NMR (242.94 MHz, C_6D_6, 295 K):

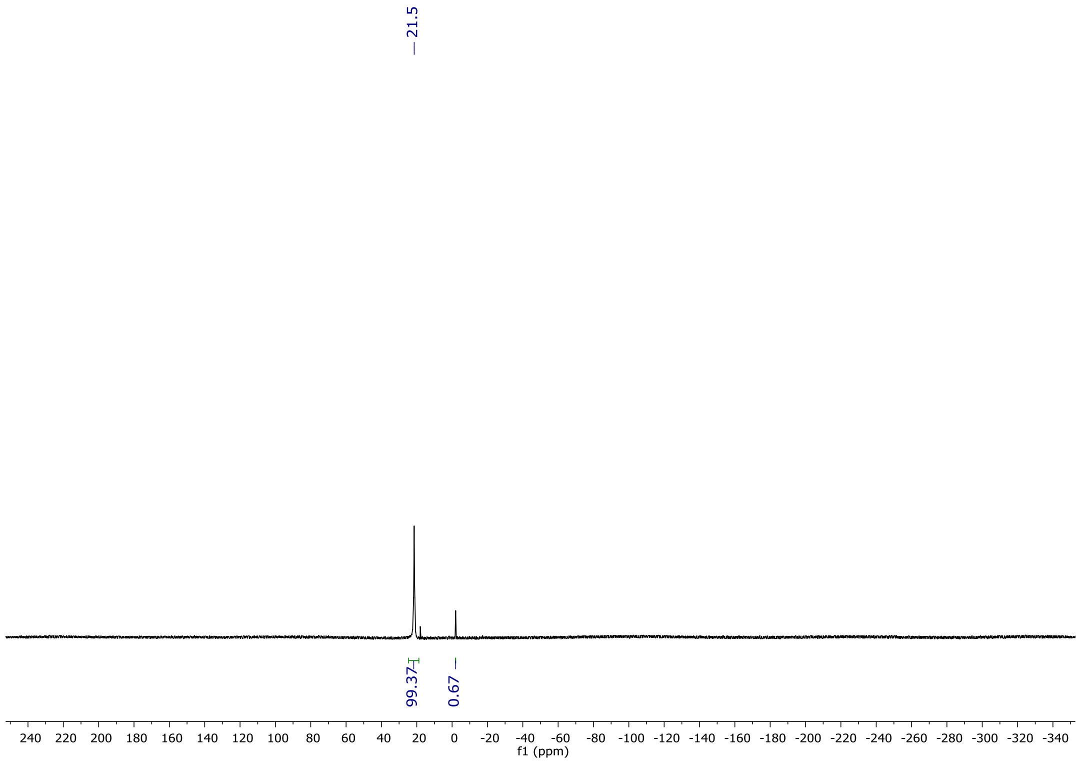

[^{iPr}(^{cbz}PNP)Zr($^{4\text{-}Me}$bipy)Cl] (3-Me):

^{1}H NMR (600.13 MHz, C_6D_6, 295 K):

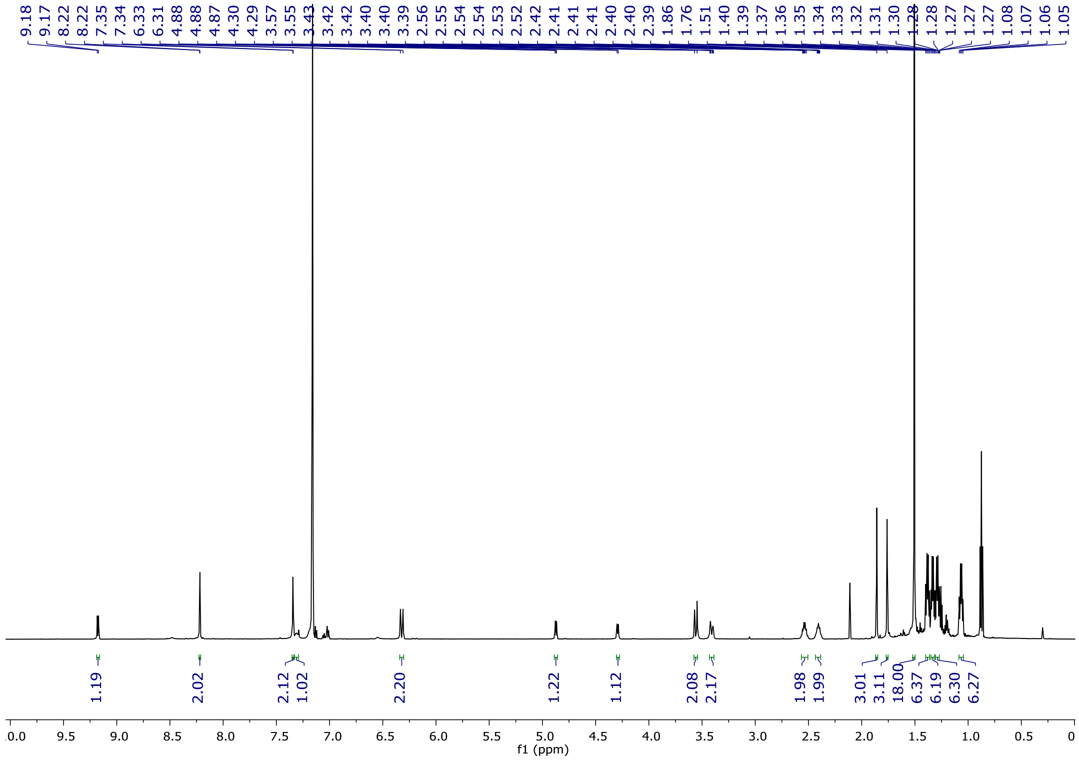

^{13}C NMR (150.90 MHz, C_6D_6, 295 K):

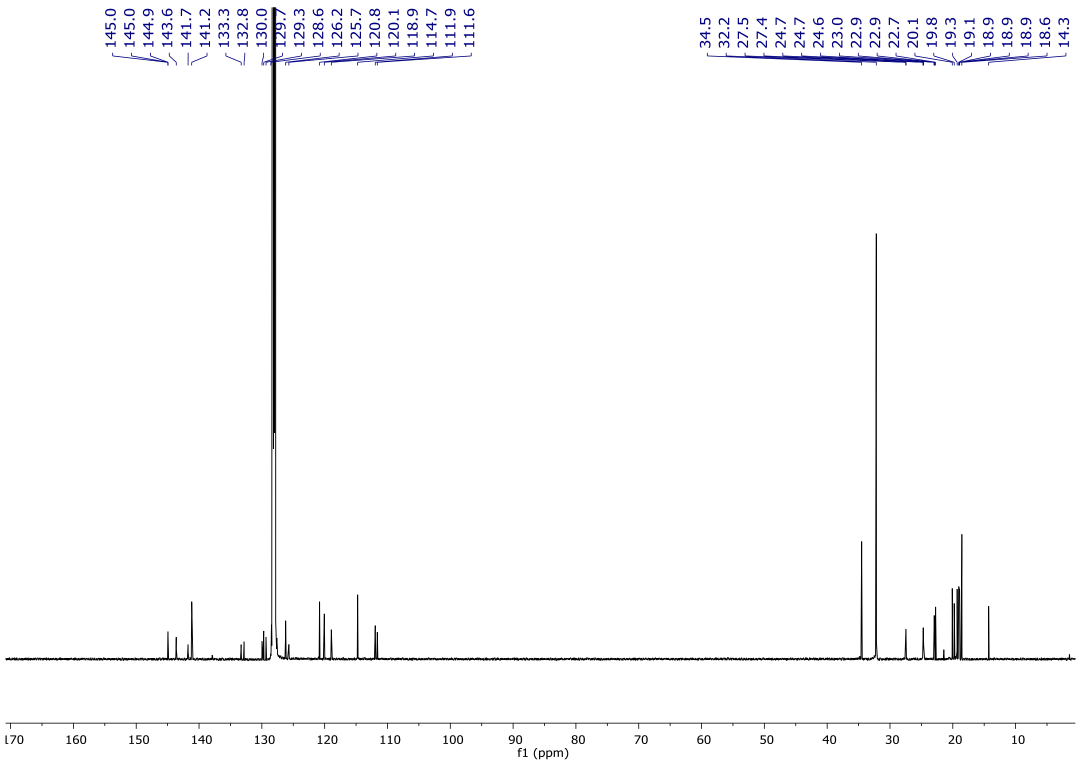

^{31}P-NMR (242.94 MHz, C_6D_6, 295 K):

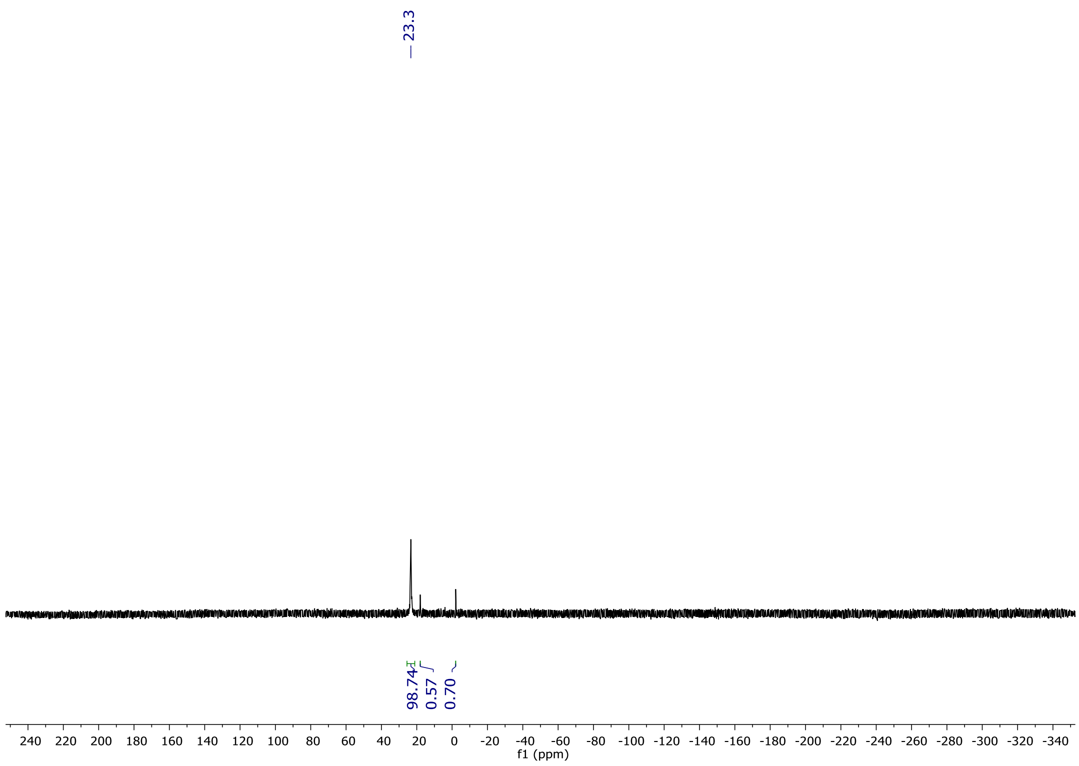

[^{iPr}(^{cbz}PNP)Zr($^{4\text{-}Et}$bipy)Cl] (3-Et):

^{1}H NMR (600.13 MHz, C_6D_6, 295 K):

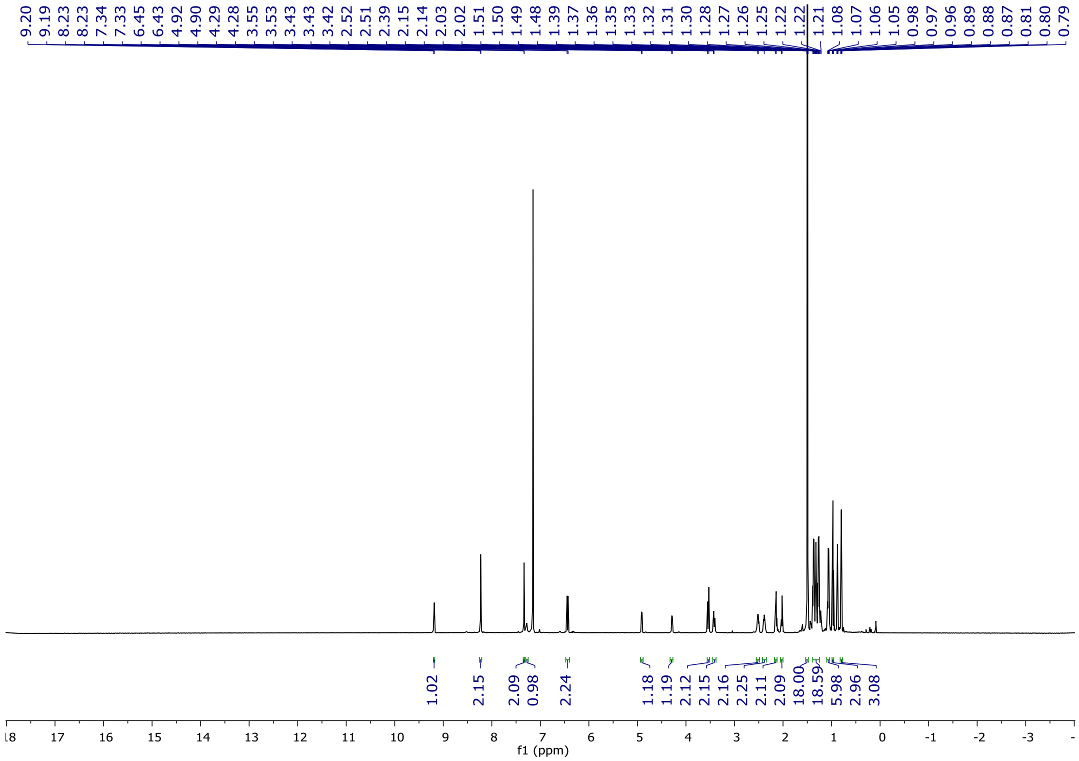

^{13}C NMR (150.90 MHz, C_6D_6, 295 K):

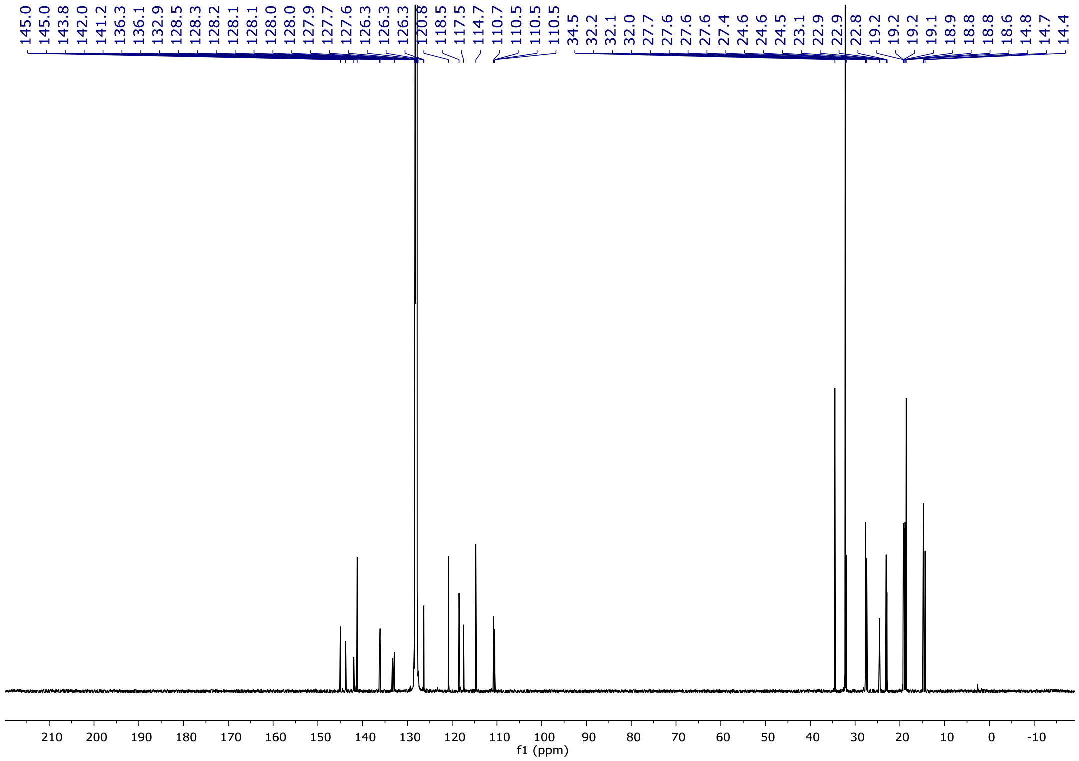

^{31}P-NMR (242.94 MHz, C_6D_6, 295 K):

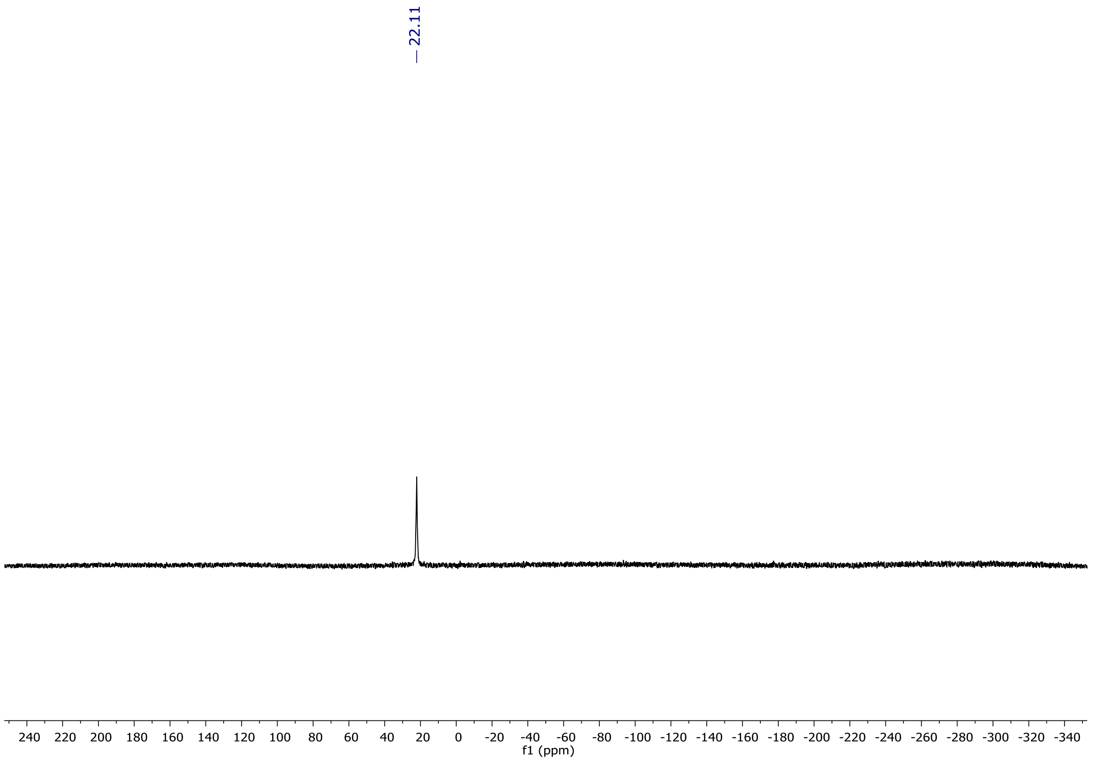

$[^{iPr}(^{cbz}PNP)Zr(^{4\text{-}tBu}bipy)Cl]$ (3-tBu):

^{1}H NMR (600.13 MHz, C_6D_6, 295 K):

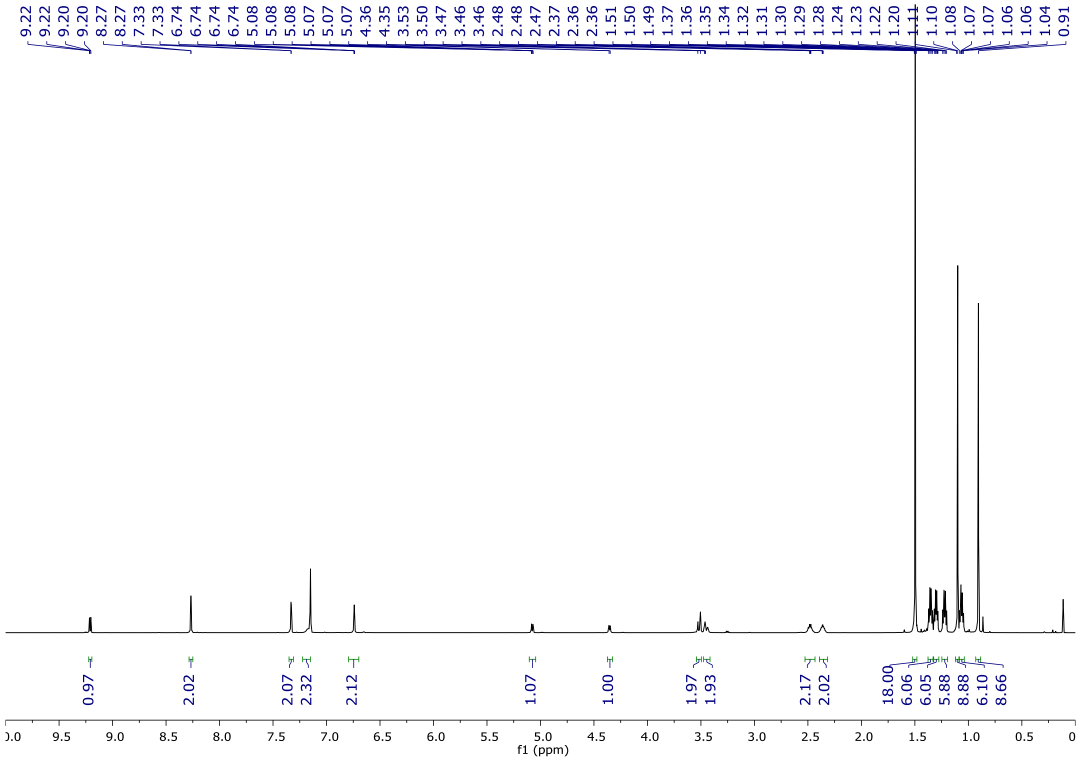

^{13}C NMR (150.90 MHz, C_6D_6, 295 K):

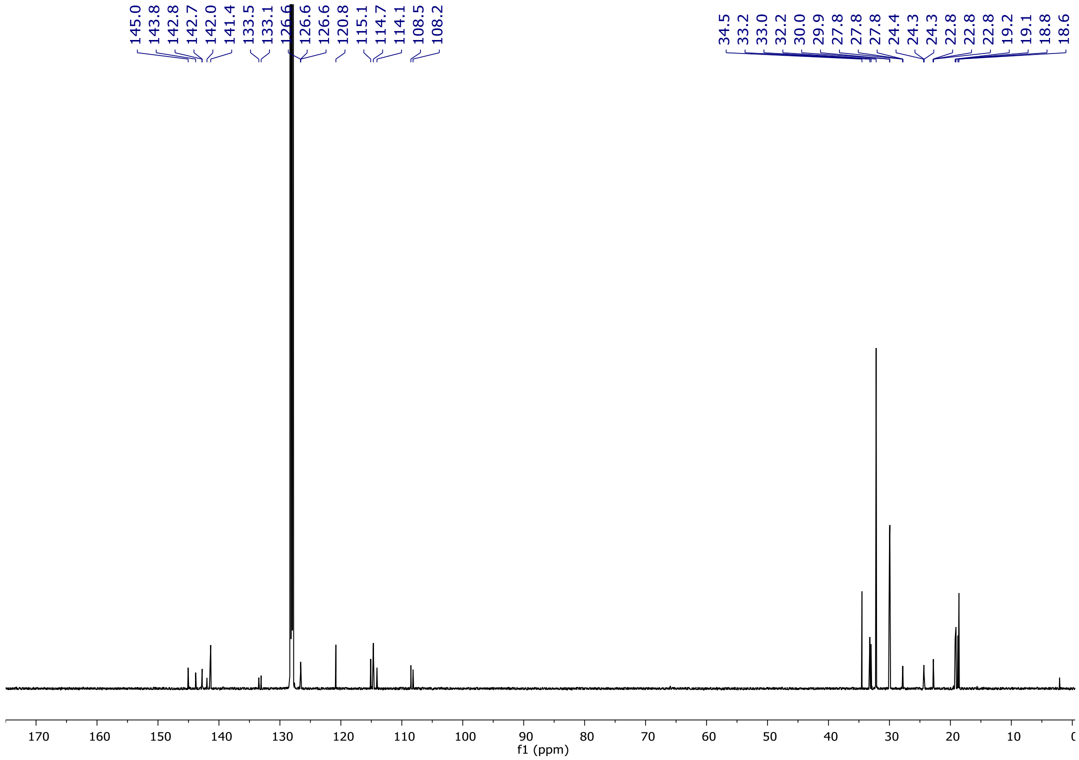

^{31}P-NMR (242.94 MHz, C_6D_6, 295 K):

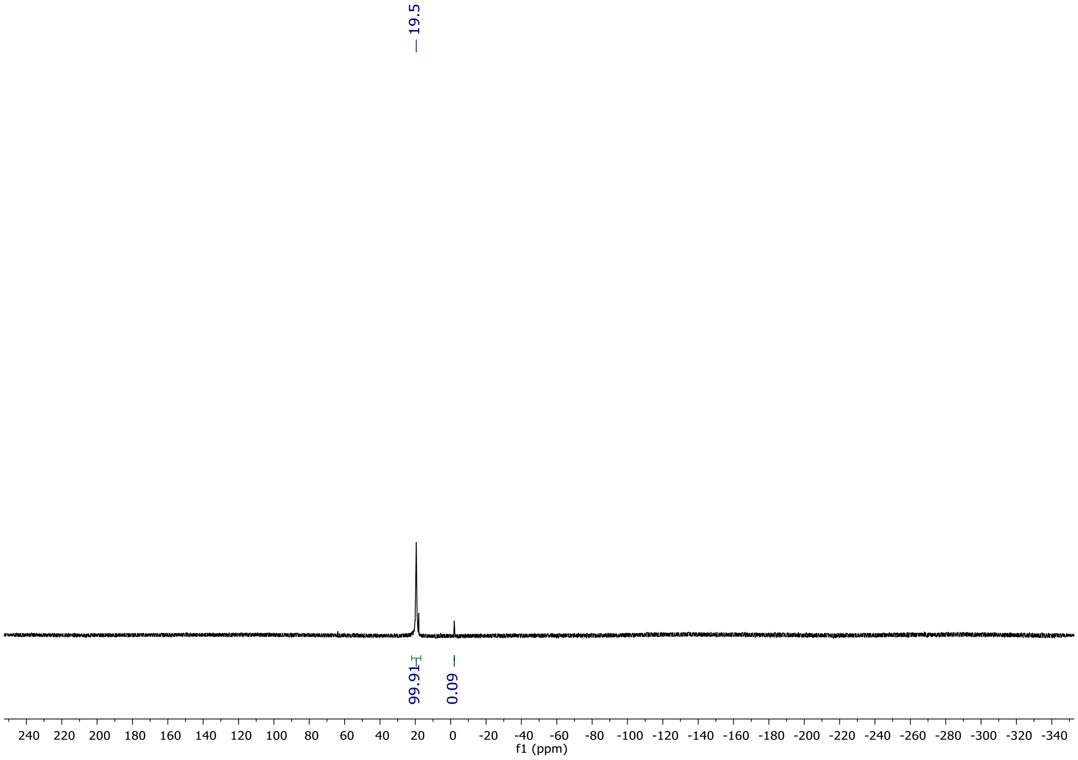

$[^{iPr}(^{cbz}\mathrm{PNP})\mathrm{Zr}(^{4\text{-}Bn}\mathrm{bipy})\mathrm{Cl}]$ (3-Bn):

^{1}H NMR (600.13 MHz, C_6D_6, 295 K):

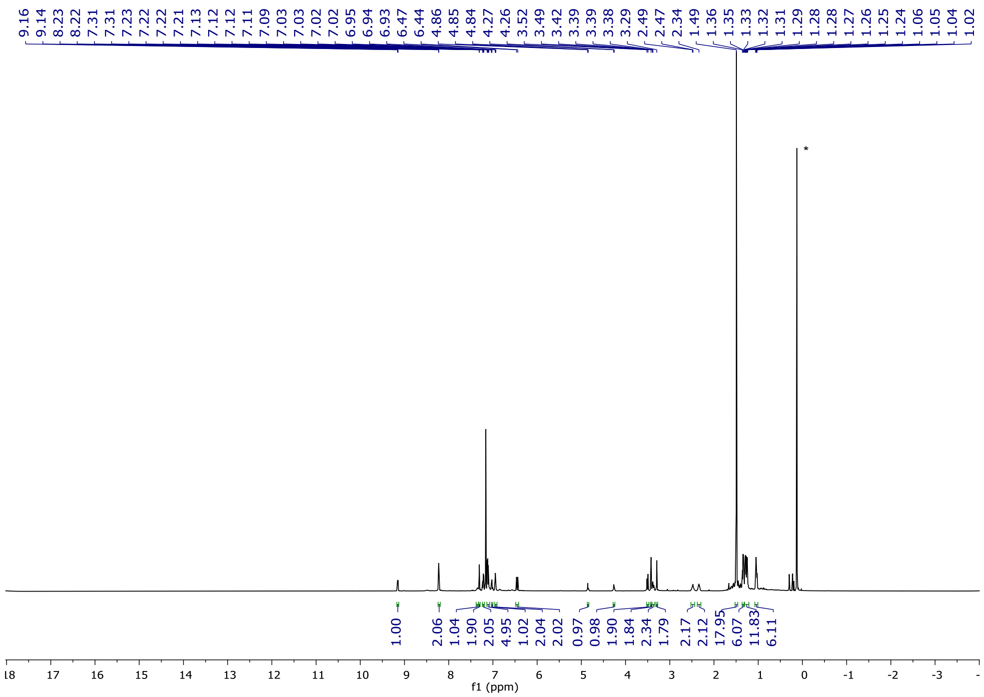

^{13}C NMR (150.90 MHz, C_6D_6, 295 K):

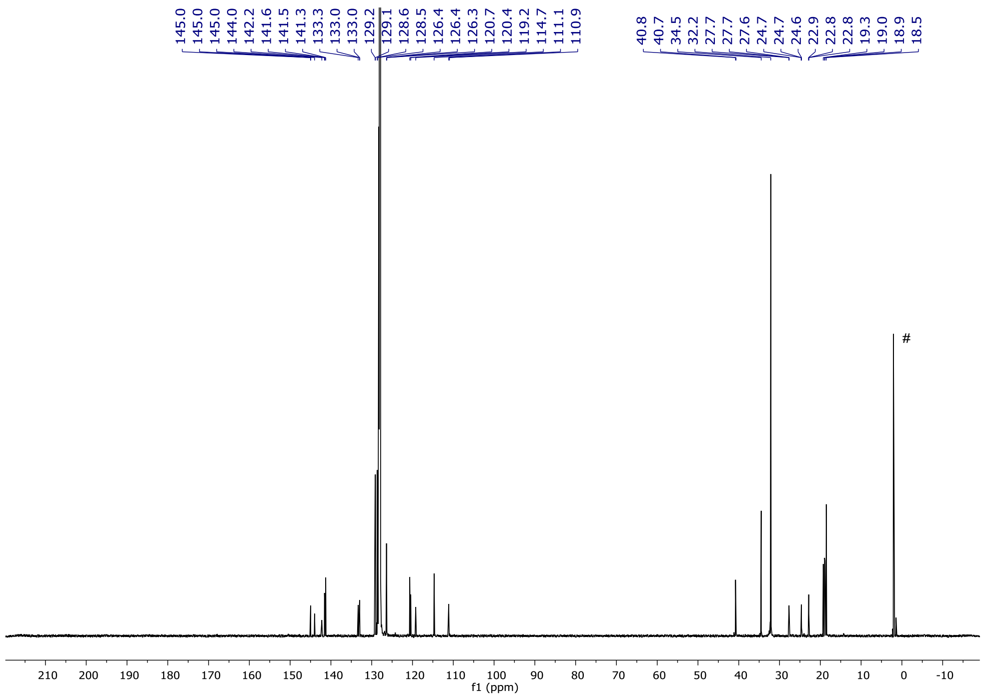

1 # indicates residual HMDSO

^{31}P-NMR (242.94 MHz, C_6D_6, 295 K):

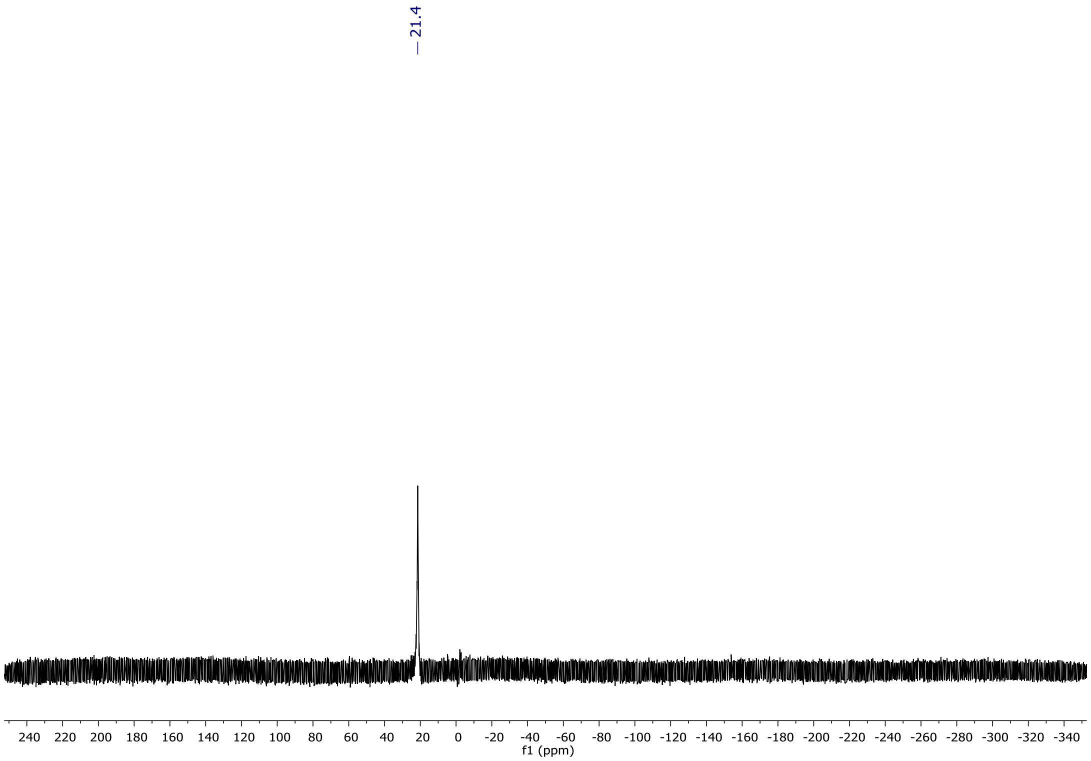

[^{iPr}(^{cbz}PNP)Zr($^{4\text{-}Ph}$bipy)Cl] (3-Ph):

^{1}H NMR (600.13 MHz, C_6D_6, 295 K):

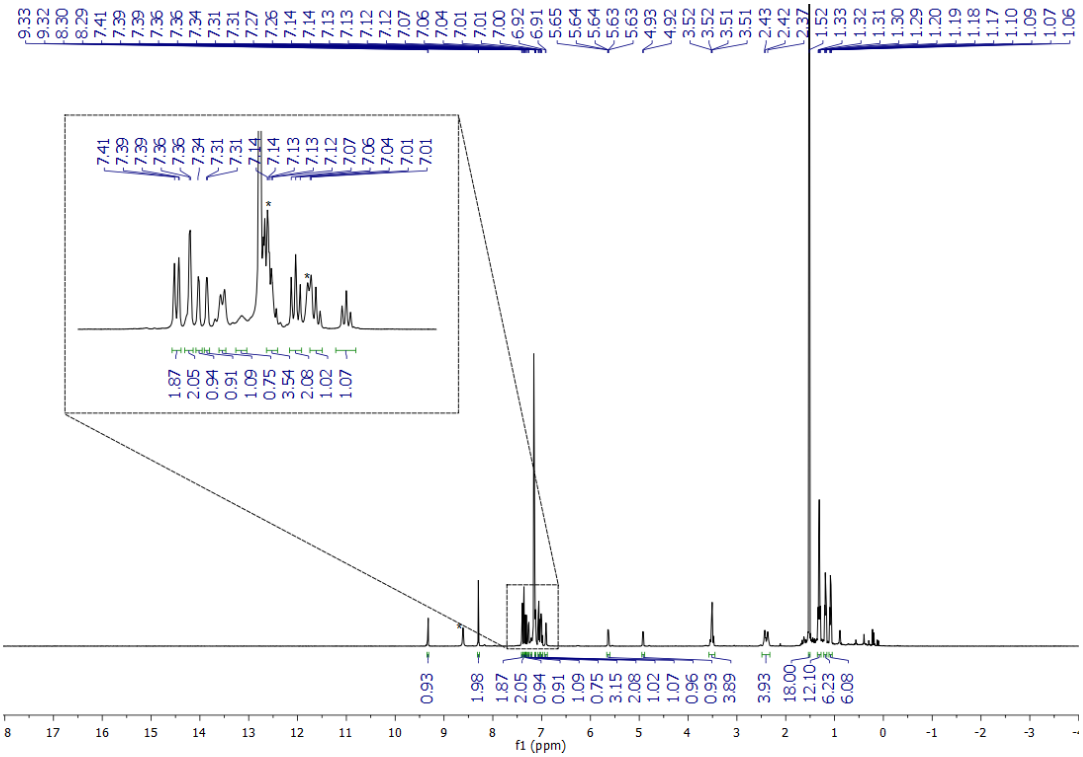

^{13}C NMR (150.90 MHz, C_6D_6, 295 K):

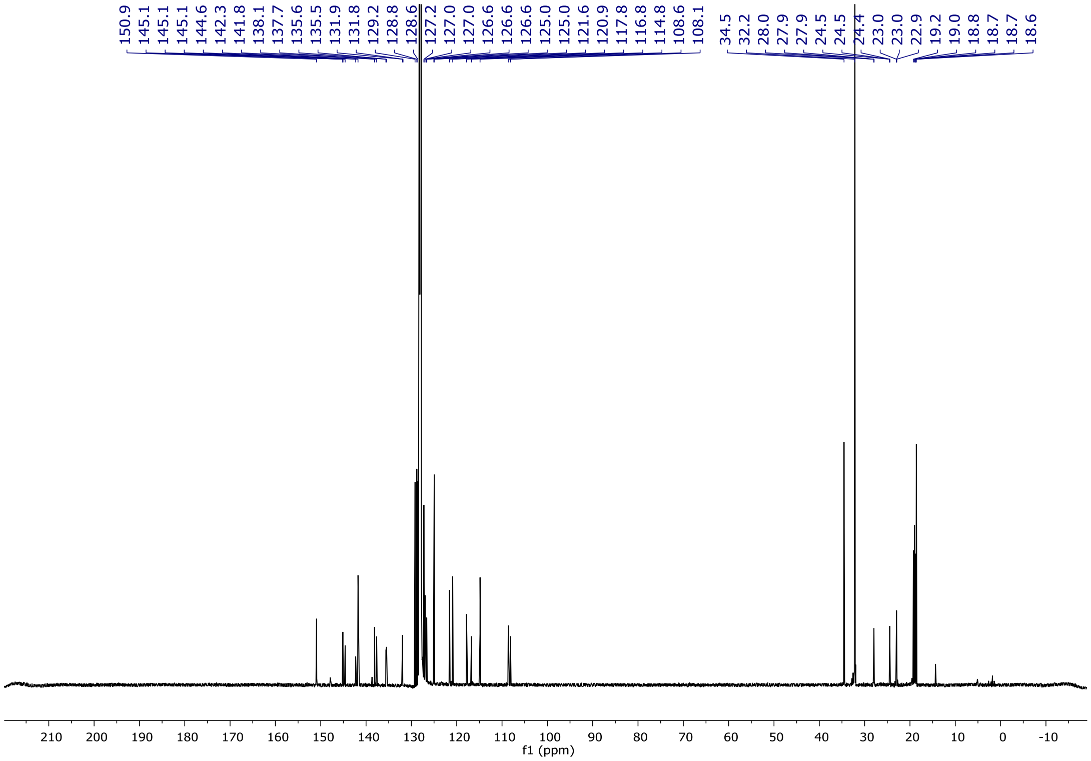

^{31}P-NMR (242.94 MHz, C_6D_6, 295 K):

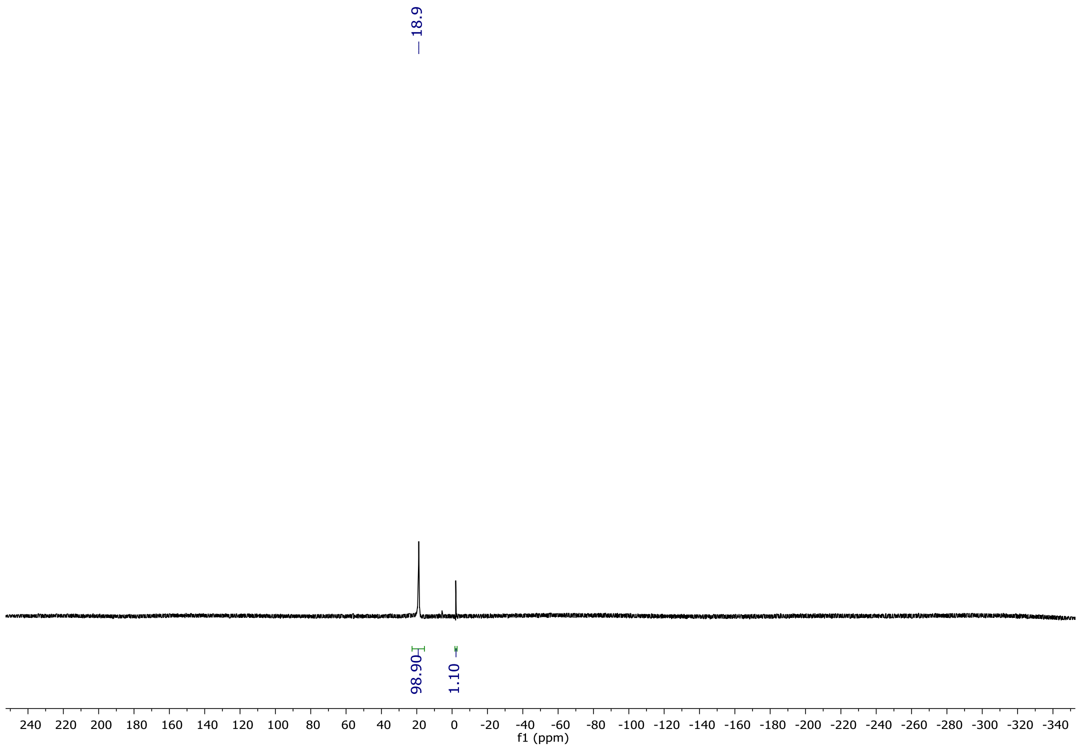

[^{iPr}(^{cbz}PNP)Zr($^{4\text{-Sty}}$bipy)Cl] (3-Sty):

^{1}H NMR (600.13 MHz, C_6D_6, 295 K):

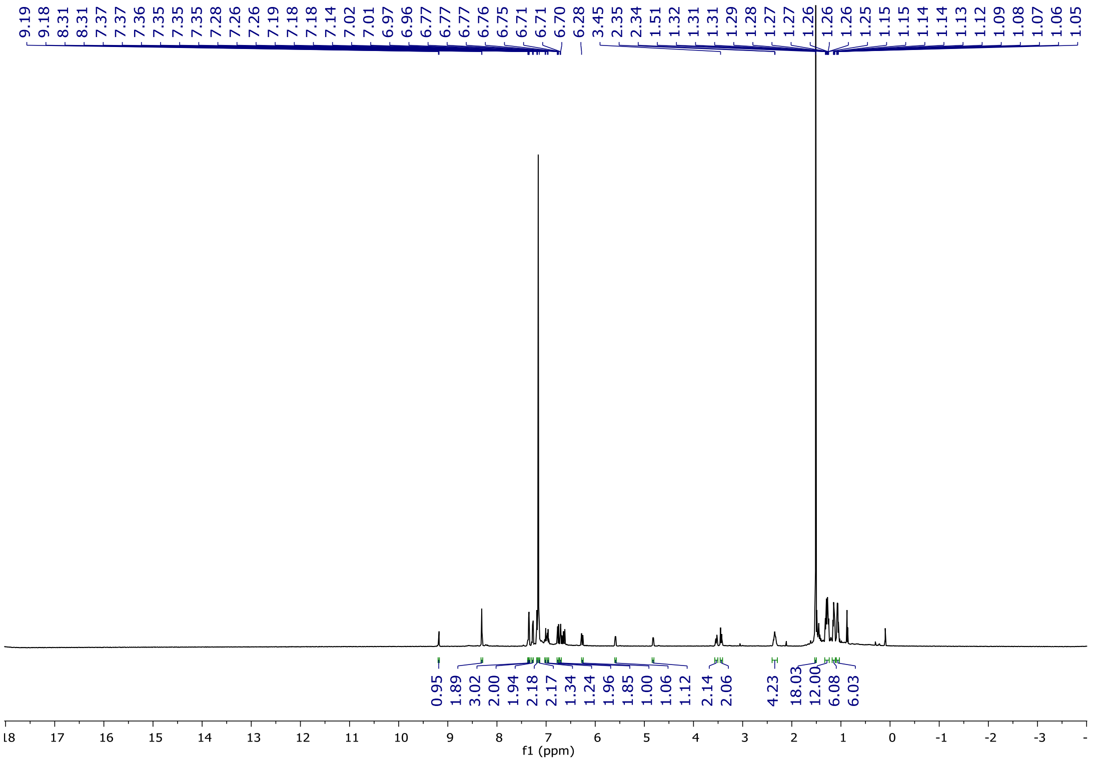

^{13}C NMR (150.90 MHz, C_6D_6, 295 K):

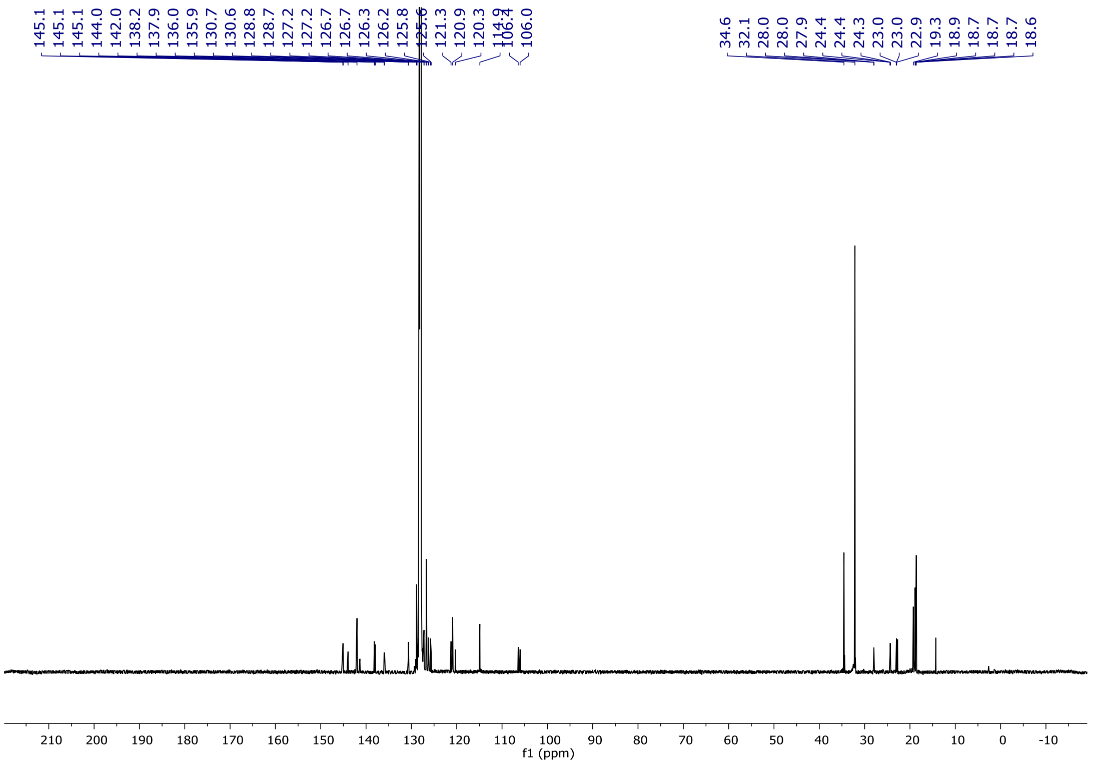

^{31}P-NMR (242.94 MHz, C_6D_6, 295 K):

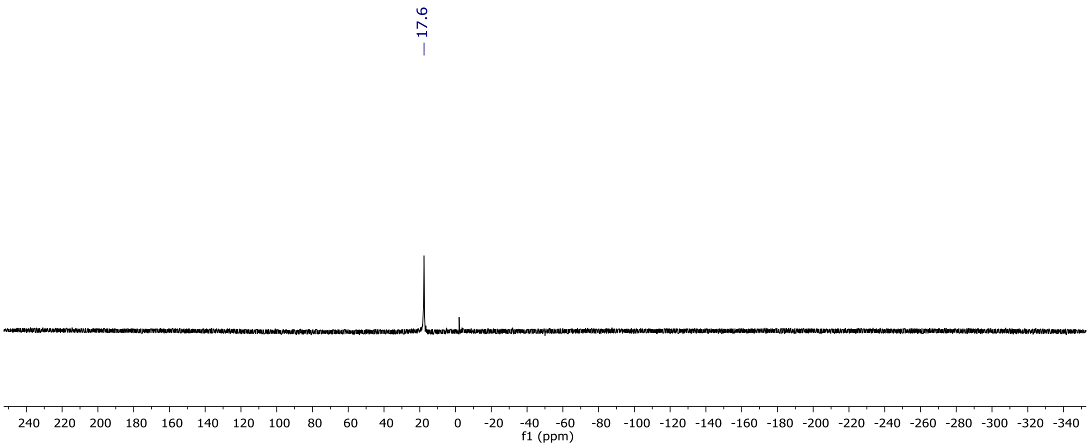

[(^{iPr}(^{cbz}PNP)Zr (μ^2-DMAP)(dmap)Cl] (4):

^{1}H NMR (600.13 MHz, C_6D_6, 295 K):

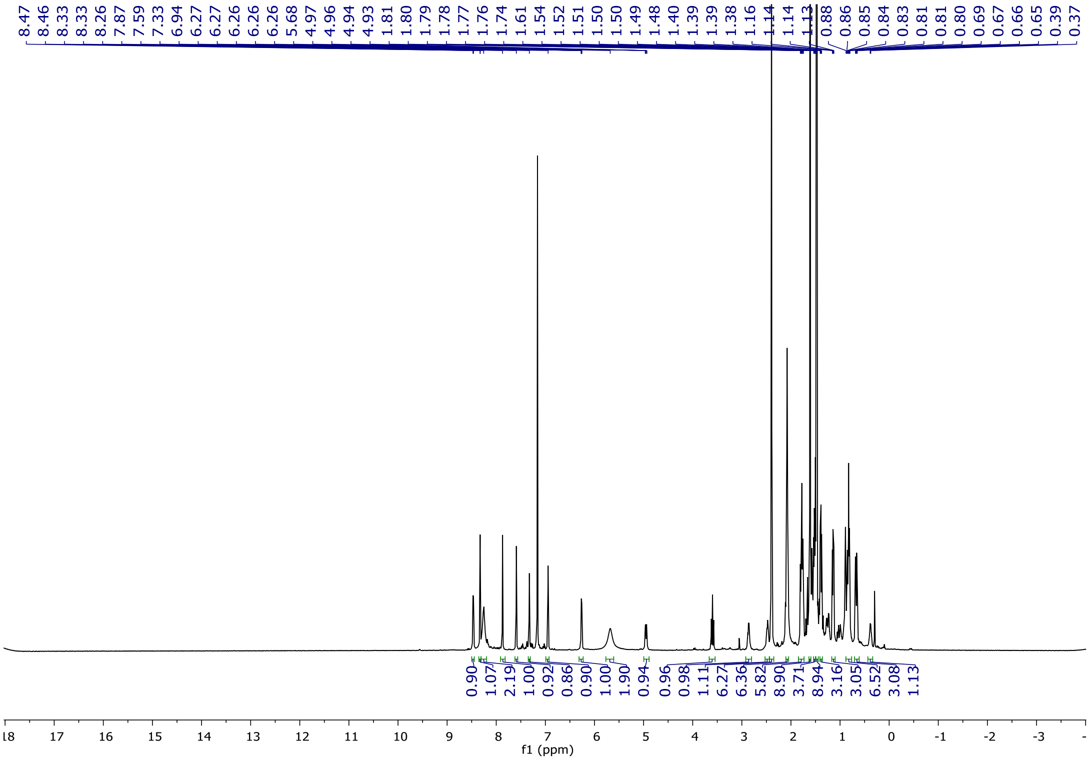

^{13}C NMR (150.90 MHz, C_6D_6, 295 K):

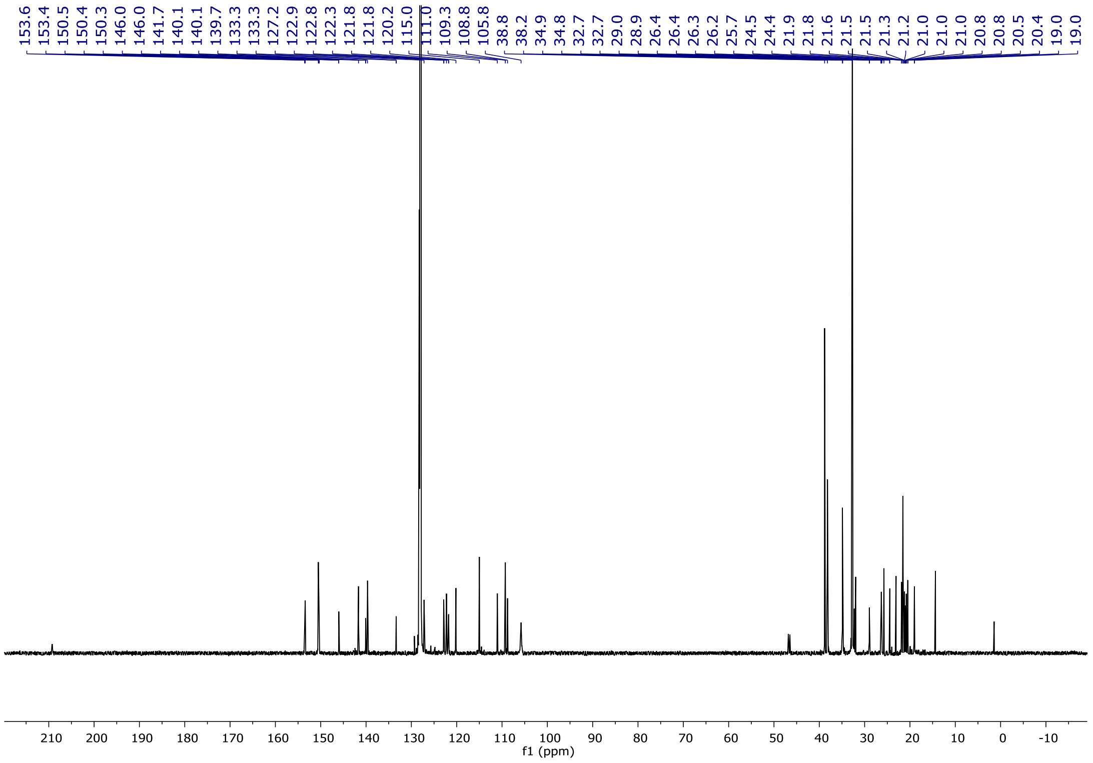

^{31}P-NMR (242.94 MHz, C_6D_6, 295 K):

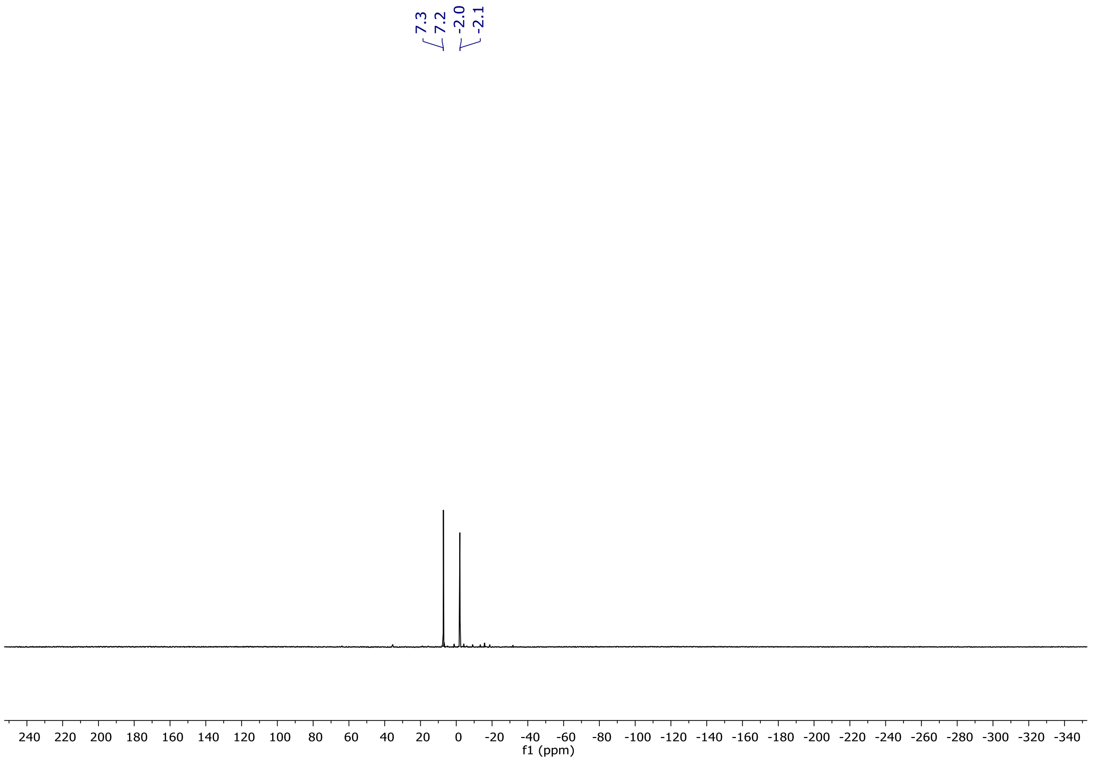

[(^{iPr}(^{cbz}PNP)Zr (bisisoquinoline)Cl] (5):

^{1}H NMR (600.13 MHz, C_6D_6, 295 K):

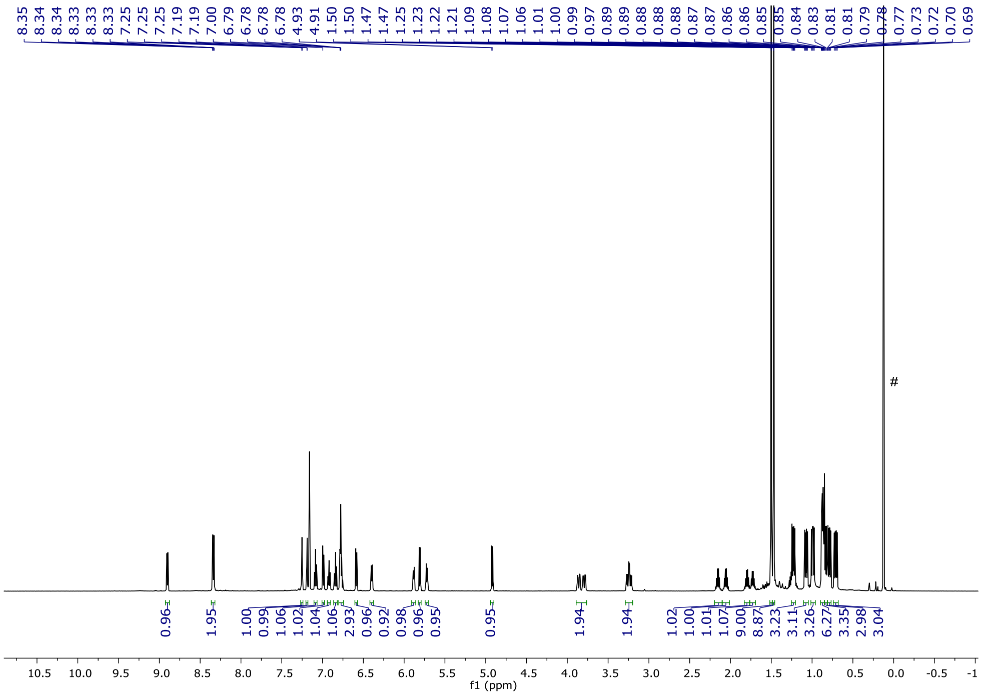

2 # indicates residual HMDSO

^{13}C NMR (150.90 MHz, C_6D_6, 295 K):

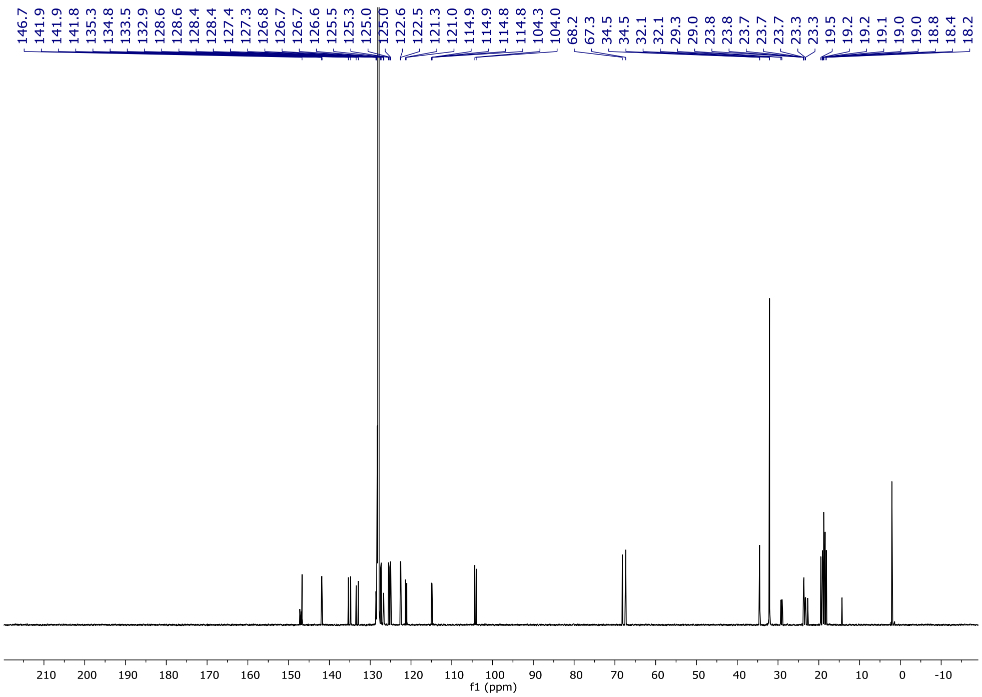

^{31}P-NMR (242.94 MHz, C_6D_6, 295 K):

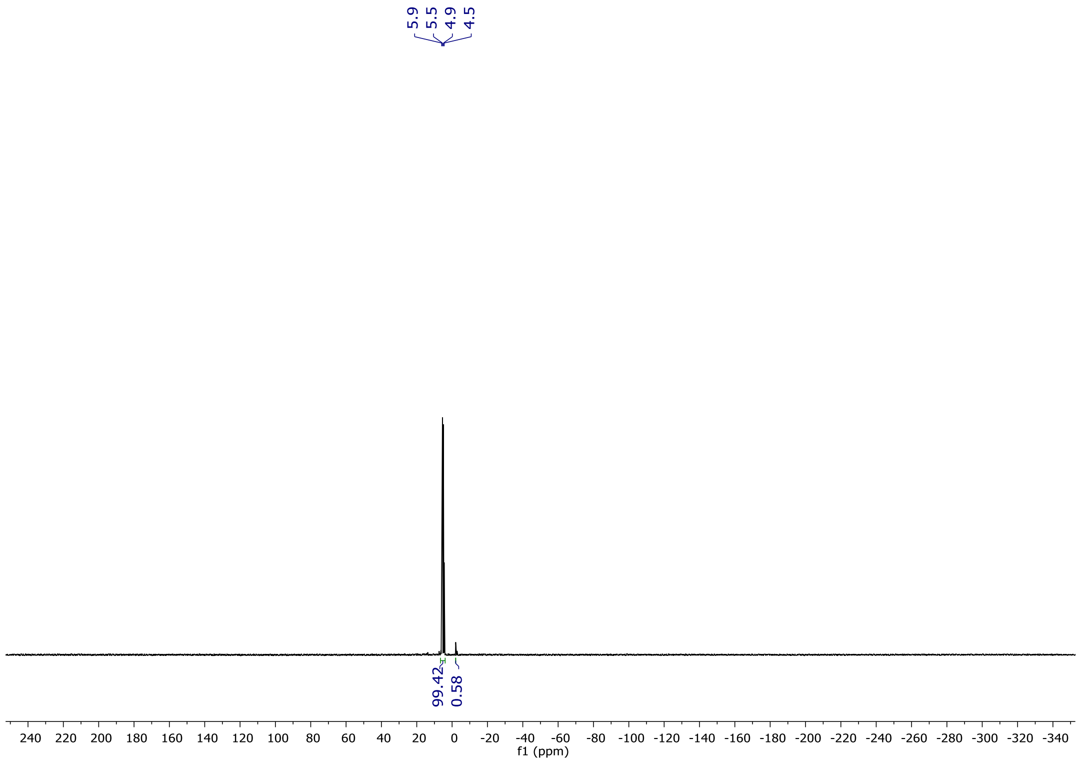
5.9
5.5
4.9
4.5
99.42
0.58
240 220 200 180 160 140 120 100 80 60 40 20 0 -20 -40 -60 -80 -100 -120 -140 -160 -180 -200 -220 -240 -260 -280 -300 -320 -340
f1 (ppm)

X-ray crystal Structure Determinations

Crystal data and details of the structure determinations are compiled in Tables S1 and S2. Full shells of intensity data were collected at low temperature with an Agilent Technologies Supernova-E CCD diffractometer (Mo- or Cu-K_α radiation, microfocus X-ray tube, multilayer mirror optics). Detector frames (typically ω-, occasionally φ-scans, scan width 0.4...1°) were integrated by profile fitting.[1,2] Data were corrected for air and detector absorption, Lorentz and polarization effects[#2] and scaled essentially by application of appropriate spherical harmonic functions.[3,4] Absorption by the crystal was treated with a semiempirical multiscan method (as part of the scaling process), (and) augmented by a spherical correction,[3,4] or numerically (Gaussian grid).[3,5] An illumination correction was performed as part of the numerical absorption correction.[3]

The structures were solved by the heavy atom method combined with structure expansion by direct methods applied to difference structure factors[6] (complex **2**) or by the charge flip procedure[7] (all other complexes) and refined by full-matrix least squares methods based on F^2 against all unique reflections.[8] All non-hydrogen atoms were given anisotropic displacement parameters. Hydrogen atoms were generally input at calculated positions and refined with a riding model. When justified by the quality of the data the positions of some hydrogen atoms were taken from difference Fourier syntheses and refined.

When found necessary, disordered groups and/or solvent molecules were subjected to suitable geometry and adp restraints.[9] Due to severe disorder and fractional occupancy, electron density attributed to solvent of crystallization (*n*-pentane and/or diethyl ether) was removed from the structure of **5** with the BYPASS procedure,[10] as implemented in PLATON (squeeze/hybrid).[11] Partial structure factors from the solvent masks were included in the refinement as separate contributions to F_{calc}.

Crystals of **2** were twinned; the structure was solved with a de-twinned partial dataset. Final refinement was carried out against all single and composite reflections involving the major component (refined twin fractions 0.66:0.34).

CCDC 1826625 - 1826628 contains the supplementary crystallographic data for this paper. These data can be obtained free of charge from The Cambridge Crystallographic Data Centre via https://www.ccdc.cam.ac.uk/data_request/cif.

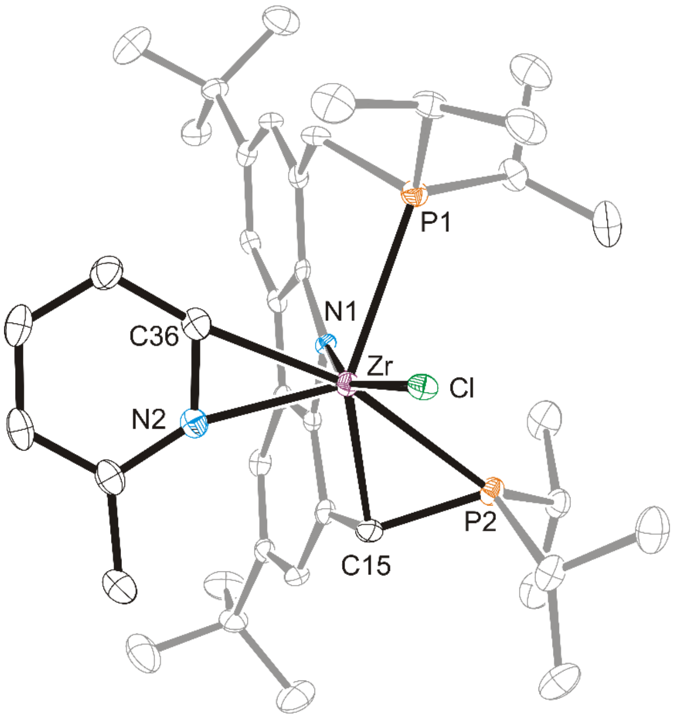

Figure S2 Molecular structure of **2**. Hydrogen atoms were omitted for clarity, ellipsoids set at 50 % probability. Selected bond lengths [Å] and angles [°] for **2**: Zr-Cl 2.4635(7), Zr-P1 2.7890(7), Zr-P2 2.7110(7), Zr-N1 2.186(2), Zr-N2 2.171(2), Zr-C15 2.386(3), Zr-C36 2.242(3), Cl-Zr-P1 87.92(2), Cl-Zr-P2 91.63(2), P2-Zr-P1 103.92(2), N1-Zr-Cl 159.49(6), N1-Zr-N2 95.34(8), N2-Zr-Cl 102.52(6), N2-Zr-P1 124.32(6), N2-Zr-P2 129.72(6).

.

Table S1 Details of the crystal structure determinations of **2** and **3-tBu**.

Compound	**2**	**3-tBu**
Formula	$C_{40}H_{59}ClN_2P_2Zr$	$C_{52}H_{78}ClN_3P_2Zr$
M_r	756.50	933.78
Crystal system	triclinic	monoclinic
Space group	P-1	$P2_1/n$
a /Å	11.9355(4)	12.10234(18)
b /Å	12.1743(4)	13.69809(19)
c /Å	15.3187(4)	31.6606(4)
α /°	112.686(3)	
β /°	97.711(2)	91.0656(14)
γ /°	98.630(3)	
V/Å^3	1984.94(11)	5247.76(13)
Z	2	4
F_{000}	800	1992
d_c /Mg·m^{-3}	1.266	1.182
X-radiation, λ /Å	Mo-$K\alpha$, 0.71073	Cu-$K\alpha$, 1.54184
μ /mm^{-1}	0.454	3.014
Absorption correction	semi-empirical	numerical
Max., min. transmission factors	1.0000, 0.7386	1.000, 0.657
Data collect. Temperat. /K	120(1)	120(1)
θ range /°	2.9 to 25.2	3.5 to 71.0
index ranges h,k,l	-14 ... 14, -14 ... 14, -18 ... 18	-14 ... 14, -16 ... 16, -38 ... 38
Reflections measured	18410	136700
Unique [R_{int}]	7999 [0.085]	10072 [0.0908]
observed [$I \geq 2\sigma(I)$]	6167	8403
$GooF$ on F^2	0.924	1.165
R indices [$F>4\sigma(F)$] $R(F)$, $wR(F^2)$	0.0360, 0.0653	0.0505, 0.1104
R indices (all data) $R(F)$, $wR(F^2)$	0.0572, 0.0684	0.0645, 0.1153
Difference density: max, min /e·Å^3	0.551, -0.458	0.854, -0.602
CCDC deposition number	1826625	1826626

Table S2 Details of the crystal structure determinations of **4**·OEt_2 and **5**·solv.

Compound	**4**·OEt_2	**5**·solv
Formula	$C_{52}H_{82}ClN_5OP_2Zr$	$C_{57}H_{80}ClN_3P_2Zr$
M_r	981.83	995.85
Crystal system	monoclinic	triclinic
Space group	$C2/c$	P-1
a /Å	37.6269(7)	12.6621(2)
b /Å	12.5284(3)	18.1002(3)
c /Å	22.9308(4)	23.9357(3)
α /°		100.8658(12)
β /°	99.4621(19)	95.4948(13)
γ /°		103.4908(14)
V /Å^3	10662.7(4)	5182.33(15)
Z	8	4
F_{000}	4192	2120
d_c /Mg·m^{-3}	1.223	1.276
X-radiation, λ /Å	Mo-$K\alpha$, 0.71073	Mo-$K\alpha$, 0.71073
μ /mm^{-1}	0.356	0.365
Absorption correction	numerical	numerical
Max., min. transmission factors	0.978, 0.963	0.983, 0.934
Data collect. Temperat. /K	120(1)	120(1)
θ range /°	2.6 to 29.6	3.1 to 32.3
index ranges h,k,l	-52 ... 52, -17 ... 17, -31 ... 31	-19 ... 18, -26 ... 27, -35 ... 35
Reflections measured	90595	184312
Unique [R_{int}]	14980 [0.1174]	35040 [0.0705]
observed [$I \geq 2\sigma(I)$]	10345	25107
$GooF$ on F^2	1.040	1.037
R indices [$F>4\sigma(F)$] $R(F)$, $wR(F^2)$	0.0539, 0.0987	0.0497, 0.1091
R indices (all data) $R(F)$, $wR(F^2)$	0.0940, 0.1124	0.0787, 0.1213
Difference density: max, min /e·Å^3	0.842, -0.541	1.906, -0.999
CCDC deposition number	1826627	1826628

UV/Vis Spectroscopy

UV/Vis absorption spectra were recorded with a Cary 5000 UV/Vis/NIR and were baseline- and solvent-corrected.

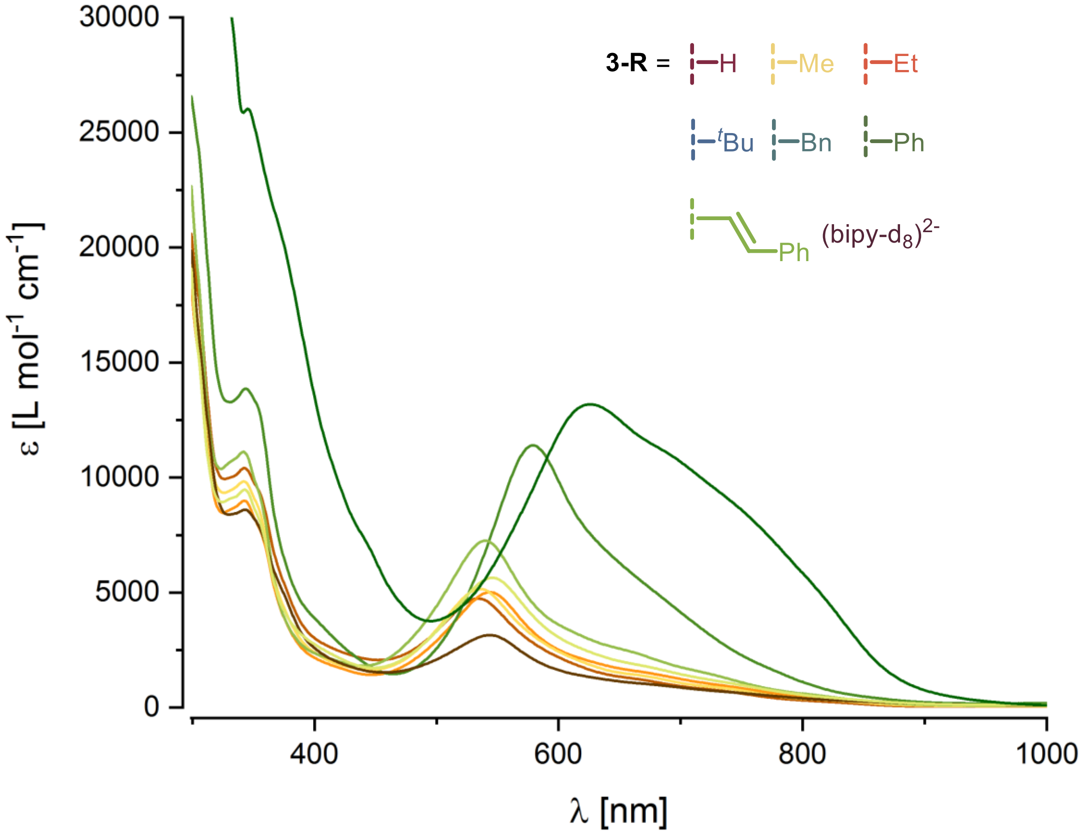

Note: We observed a gradual decomposition of the product complexes during UV/Vis analysis, which we contribute to the low concentrations and possibly to residual water traces. Therefore, we did not determine extinction coefficients and view the UV/Vis data as qualitative results, only.

DFT Calculations

Computational Details

Geometry optimizations have been performed using Guassian 09, Revision D01[12] at the PBE0 level of hybrid density functional theory,[13] with inclusion of D3(bj) corrections in the optimization process.[14,15] The geometry of all the structures optimized is available as a single xyz file alongside the Supporting Information. The atoms C, H, N, P and Cl were represented by an svp basis set.[16] The Zr atom was represented by Dolg's pseudo potential and the associated basis set.[17,18] The solvent (benzene) influence was taken into consideration through single-point calculations on the gas-phase optimized geometries with SCRF calculations within the SMD model.[19] For the SCRF calculations, the atoms were treated with a def2-qzvp basis set.[20] All energies reported are Gibbs free energies obtained by summing the SMD energy (including D3 corrections) and the gas phase Gibbs contribution at 333 K and 1 atm (cf Table S3 below).

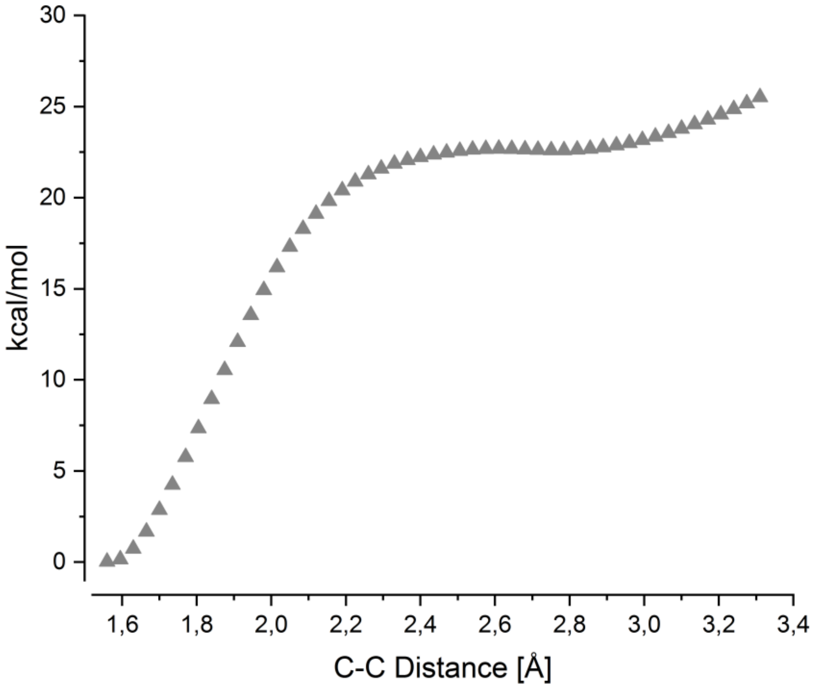

Figure S3: Potential energy surface scan along the C-C distance between C1 and C1' isoquinoline carbon atoms to form complex **5**.

Table S3: SMD Energies (a.u.) and Gibbs correction (a.u.) used to compute the Gibbs free energy of all the molecules presented in this work.

Species	E(SMD/Def2-QZVP)	δG (333 K)
Toluene	-271,36448	0,09217
Pyridine	-248,10619	0,05822
2-picoline	-287,39798	0,08329
DMAP	-381,99049	0,12396
Isoquinoline	-401,6431	0,1008
H_2	-1,1685413	-0,0033
1	-2843,1839	0,84371
1-py	-2819,909398	0,796444
CH-1	-2819,92350291	0,800803
CH-1-TS	-2819,89829593	0,794394
CH-2	-2819,91198103	0,794476
CH-3	-3068,02924336	0,882498
CH-3-TS	-3067,98681767	0,879403
CH-4	-3068,05523714	0,885461
CH-4-TS	-3068,00852369	0,879221
3-H	-3068,07189707	0,871924
Syn-1	-3068,01812485	0,882529
Syn-1-TS	-3068,00681934	0,88508
Syn-2	-3068,05855031	0,888396
Syn-3	-3068,03767099	0,884922
Syn-3-TS	-3068,02694763	0,88175
Syn-4	-3068,05397681	0,883937
Syn-4-TS	-3068,02933803	0,880007
Anti-1	-3068,03868842	0,884396
Anti-1-TS	-3068,02826862	0,885277
Anti-2	-3068,06510873	0,887215
Anti-3	-3068,03921035	0,888
Anti-3-TS	-3068,02581145	0,883434
Anti-4	-3068,05275074	0,884267
Anti-4-TS	-3067,9879895	0,879034
Anti-5	-3066,86211505	0,86424
1-^{Me}py	-2859,2038707	0,824096
^{Me}CH-1	-2859,21075773	0,826225
^{Me}CH-1-TS	-2859,19088347	0,815687
^{Me}CH-2	-2859,20341863	0,820255
^{Me}Cy-1	-2859,20924737	0,821276
^{Me}Cy-1-TS	-2859,16721051	0,819656
^{Me}Cy-2	-2858,01829686	0,802108
^{Me}Cy-3	-3145,42807742	0,91269
^{Me}Cy-3-TS	-3145,38295967	0,916795
^{Me}Cy-4	-3145,43135824	0,918265
^{Me}Syn-1	-3146,60611701	0,937909
^{Me}Syn-1-TS	-3146,58404591	0,940131
^{Me}Syn-2	-3146,60300321	0,942183
^{Me}Anti-1	-3146,61042078	0,940597
^{Me}Anti-1-TS	-3146,59582	0,940929
^{Me}Anti-2	-3146,61682768	0,940383
1-^{DMAP}py	-2953,78165178	0,86171
^{DMAP}CH-1	-2953,782211	0,86126

^{DMAP}**CH-1-TS**	-2953,78264006	0,860233
^{DMAP}**CH-2**	-2953,79849655	0,859979
^{DMAP}**Cy-1**	-2953,80067191	0,861221
^{DMAP}**Cy-1-TS**	-2953,75879302	0,857594
^{DMAP}**Cy-2**	-2952,61534278	0,84427
^{DMAP}**Cy-3**	-3334,62424984	0,993452
^{DMAP}**Cy-3-TS**	-3334,57762441	0,994707
^{DMAP}**Cy-4**	-3334,64282082	0,995532
^{DMAP}**Syn-1**	-3335,79344648	1,014842
^{DMAP}**Syn-1-TS**	-3335,75979844	1,016674
^{DMAP}**Syn-2**	-3335,81269041	1,018854
^{DMAP}**Anti-1**	-3335,79800544	1,016423
^{DMAP}**Anti-1-TS**	-3335,78617401	1,018118
^{DMAP}**Anti-2**	-3335,81980431	1,016745
^{IQ}**Syn-1**	-3375,1378415	0,972502
^{IQ}**Syn-1-TS**	-3375,10501471	0,972243
^{IQ}**Syn-2**	-3375,16589291	0,975655
^{IQ}**Anti-1**	-3375,12927968	0,972485
^{IQ}**Anti-1-TS**		
^{IQ}**Anti-2**	-3375,16401606	0,975875
**Syn-1T**	-3068.03175710	0,882472

References

1 K. Kabsch, in: M. G. Rossmann, E. Arnold (eds.), *"International Tables for Crystallography" Vol. F*, Ch. 11.3, Kluwer Academic Publishers, Dordrecht, 2001.

2 *CrysAlisPro*, Agilent Technologies UK Ltd., Oxford, UK 2011-2014 and Rigaku Oxford Diffraction, Rigaku Polska Sp.z o.o., Wrocław, Poland, 2015-2016.

3 *SCALE3 ABSPACK, CrysAlisPro*, Agilent Technologies UK Ltd., Oxford, UK 2011-2014 and Rigaku Oxford Diffraction, Rigaku Polska Sp.z o.o., Wrocław, Poland, 2015-2016.

4 R. H. Blessing, *Acta Cryst.* 1995, ***A51***, 33.

5 W. R. Busing, H. A. Levy, *Acta Cryst.* 1957, ***10***, 180.

6 (a) P. T. Beurskens, G. Beurskens, R. de Gelder, J. M. M. Smits, S. Garcia-Granda, R. O. Gould, *DIRDIF-2008*, Radboud University Nijmegen, The Netherlands, 2008; (b) P. T. Beurskens, in: G. M. Sheldrick, C. Krüger, R. Goddard (eds.), *Crystallographic Computing 3*, Clarendon Press, Oxford, UK, 1985, p. 216.

7 (a) L. Palatinus, *SUPERFLIP*, EPF Lausanne, Switzerland and Fyzikální ústav AV ČR, v. v. i., Prague, Czech Republic, 2007-2014; (b) L. Palatinus, G. Chapuis, *J. Appl. Cryst.* 2007, **40**, 786.

8 (a) G. M. Sheldrick, *SHELXL-20xx*, University of Göttingen and Bruker AXS GmbH, Karlsruhe, Germany 2012-2017; (b) G. M. Sheldrick, *Acta Cryst.* 2008, ***A64***, 112; (c) G. M. Sheldrick, *Acta Cryst.* 2015, ***C71***, 3.

9 P. Müller, R. Herbst-Irmer, A. L. Spek, T. R.Schneider, M. R. Sawaya in: P. Müller (ed.) *"Crystal Structure Refinement"*, Ch. 5, Oxford University Press, Oxford, 2006.

10 (a) P. v. d. Sluis, A. L. Spek, *Acta Cryst.* 1990, ***A46***, 194; (b) A. L. Spek, *Acta Cryst.* 2015, ***C71***, 9.

11 (a) A. L. Spek, *PLATON*, Utrecht University, The Netherlands; (b) A. L. Spek, *J. Appl. Cryst.* **2003**, *36*, 7.

12 Gaussian 09, Revision D.01, M. J. Frisch, G. W. Trucks, H. B. Schlegel, G. E. Scuseria, M. A. Robb, J. R. Cheeseman, G. Scalmani, V. Barone, B. Mennucci, G. A. Petersson, H. Nakatsuji, M. Caricato, X. Li, H. P. Hratchian, A. F. Izmaylov, J. Bloino, G. Zheng, J. L. Sonnenberg, M. Hada, M. Ehara, K. Toyota, R. Fukuda, J. Hasegawa, M. Ishida, T. Nakajima, Y. Honda, O. Kitao, H. Nakai, T. Vreven, J. A. Montgomery, Jr., J. E. Peralta, F. Ogliaro, M. Bearpark, J. J. Heyd, E. Brothers, K. N. Kudin, V. N. Staroverov, T. Keith, R. Kobayashi, J. Normand, K. Raghavachari, A. Rendell, J. C. Burant, S. S. Iyengar, J. Tomasi, M. Cossi, N. Rega, J. M. Millam, M. Klene, J. E. Knox, J. B. Cross, V. Bakken, C. Adamo, J. Jaramillo, R. Gomperts, R. E. Stratmann, O. Yazyev, A. J. Austin, R. Cammi, C. Pomelli, J. W. Ochterski, R. L. Martin, K. Morokuma, V. G. Zakrzewski, G. A. Voth, P. Salvador, J. J. Dannenberg, S. Dapprich, A. D. Daniels, O. Farkas, J. B. Foresman, J. V. Ortiz, J. Cioslowski, and D. J. Fox, Gaussian, Inc., Wallingford CT, 2013.

13 C. Adamo and V. Barone, *J. Chem. Phys.*, 1999, **110**, 6158–6170.

14 S. Grimme, J. Antony, S. Ehrlich and H. Krieg, *J. Chem. Phys.*, 2010, **132**, 154104.

15 S. Grimme, S. Ehrlich and L. Goerigk, *J. Comput. Chem.*, 2011, **32**, 1456–1465.

16 A. Schäfer, H. Horn and R. Ahlrichs, *J. Chem. Phys.*, 1992, **97**, 2571–2577.

17 D. Andrae, U. Häußermann, M. Dolg, H. Stoll and H. Preuß, *Theor. Chim. Acta*, 1990, **77**, 123–141.

18 K. A. Peterson, D. Figgen, E. Goll, H. Stoll and M. Dolg, *J. Chem. Phys.*, 2003, **119**, 11113–11123.

19 A. V. Marenich, C. J. Cramer and D. G. Truhlar, *J. Phys. Chem. B*, 2009, **113**, 6378–6396.

20 F. Weigend and R. Ahlrichs, *Phys. Chem. Chem. Phys.*, 2005, **7**, 3297.
